# Infimal Convolution Regularisation Functionals of BV and $\varvec{\mathrm {L}}^{\varvec{p}}$ Spaces: Part I: The Finite $\varvec{p}$ Case

**DOI:** 10.1007/s10851-015-0624-6

**Published:** 2016-02-03

**Authors:** Martin Burger, Konstantinos Papafitsoros, Evangelos Papoutsellis, Carola-Bibiane Schönlieb

**Affiliations:** Institute for Computational and Applied Mathematics, University of Münster, Münster, Germany; Institute for Mathematics, Humboldt University of Berlin, Berlin, Germany; Department of Applied Mathematics and Theoretical Physics, University of Cambridge, Cambridge, UK

**Keywords:** Total Variation, Infimal convolution, Denoising, Staircasing, $\mathrm {L}^{p}$ norms, Image decomposition

## Abstract

We study a general class of infimal convolution type regularisation functionals suitable for applications in image processing. These functionals incorporate a combination of the total variation seminorm and $\mathrm {L}^{p}$ norms. A unified well-posedness analysis is presented and a detailed study of the one-dimensional model is performed, by computing exact solutions for the corresponding denoising problem and the case $p=2$. Furthermore, the dependency of the regularisation properties of this infimal convolution approach to the choice of *p* is studied. It turns out that in the case $p=2$ this regulariser is equivalent to the Huber-type variant of total variation regularisation. We provide numerical examples for image decomposition as well as for image denoising. We show that our model is capable of eliminating the staircasing effect, a well-known disadvantage of total variation regularisation. Moreover as *p* increases we obtain almost piecewise affine reconstructions, leading also to a better preservation of hat-like structures.

## Introduction

In this paper, we introduce a family of novel $\mathrm {TV}$–$\mathrm {L}^{p}$ infimal convolution type functionals with applications in image processing:

1.1 $$\begin{aligned}&\mathrm {TVL}_{\alpha ,\beta }^{p}(u):=\inf _{w\in \mathrm {L}^{p}(\Omega )} \alpha \Vert Du-w\Vert _{\mathcal {M}}+\beta \Vert w\Vert _{\mathrm {L}^{p}(\Omega )},\nonumber \\&\qquad \quad \alpha ,\beta >0 \quad \text {and} \quad p>1. \end{aligned}$$

Here $\Vert \cdot \Vert _{\mathcal {M}}$ denotes the Radon norm of a measure. The functional (1.1) is suitable to be used as a regulariser in the context of variational non-smooth regularisation in imaging applications. We study the properties of (1.1), its regularising mechanism for different values of *p* and apply it successfully to image denoising.

### Context

After the introduction of the total variation ($\mathrm {TV}$) for image reconstruction purposes [38], the use of non-smooth regularisers has become increasingly popular during the last decades (cf. [7]). They are typically used in the context of variational regularisation, where the reconstructed image is obtained as a solution of a minimisation problem of the type:

1.2 $$\begin{aligned} \min _{u} \frac{1}{s}\Vert f-Tu\Vert _{\mathrm {L}^{s}(\Omega )}^{s}+\Psi (u). \end{aligned}$$

The *regulariser* is denoted here by $\Psi$. We assume that the data *f*, defined on an open, bounded and connected domain $\Omega \subset \mathbb {R}^{2}$, have been corrupted through a bounded, linear operator *T* and additive (random) noise. Different values of *s* can be considered for the first term of (1.2), the *fidelity term*. For example, models incorporating an $\mathrm {L}^{2}$ fidelity term (resp. $\mathrm {L}^{1}$) have been shown to be efficient for the restoration of images corrupted by Gaussian noise (resp. impulse noise). Of course, other types of noise can also be considered and in those cases the form of the fidelity term is adjusted accordingly. Typically, one or more parameters within $\Psi$ balance the strength of regularisation against the fidelity term in the minimisation (1.2).

The advantage of using non-smooth regularisers is that the regularised images have sharp edges (discontinuities). For instance, it is a well-known fact that $\mathrm {TV}$ regularisation promotes piecewise constant reconstructions, thus preserving discontinuities. However, this also leads to blocky-like artefacts in the reconstructed image, an effect known as *staircasing*. Recall at this point that for two-dimensional images $u\in \mathrm {L}^{1}(\Omega )$, the definition of the total variation functional reads

1.3 $$\begin{aligned} \mathrm {TV}(u):=\sup \left\{ \int _{\Omega }u\, \mathrm {div}\phi \,\mathrm{d}x: \phi \in C_{c}^{\infty }(\Omega ,\mathbb {R}^{2}),\;\Vert \phi \Vert _{\infty }\le 1 \right\} . \end{aligned}$$

The total variation uses only first-order derivative information in the regularisation process. This can be seen from the fact that for $\mathrm {TV}(u)<\infty$ the distributional derivative *Du* is a finite Radon measure and $\mathrm {TV}(u)=\Vert Du\Vert _{\mathcal {M}}$. Moreover if $u\in \mathrm {W}^{1,1}(\Omega )$ then $\mathrm {TV}(u)=\int _{\Omega }|\nabla u|\,\mathrm{d}x$, i.e. the total variation is the $\mathrm {L}^{1}$ norm of the gradient of *u*. Higher-order extensions of the total variation functional are widely explored in the literature e.g. [4, 5, 9, 11, 12, 27, 29, 30, 34]. The incorporation of second-order derivatives is shown to reduce or even eliminate the staircasing effect. The most successful regulariser of this kind is the second-order total generalised variation (TGV) introduced by Bredies et al. [5]. Its definition reads

1.4 $$\begin{aligned} \mathrm {TGV}_{\alpha ,\beta }^{2}(u):=\min _{w\in \mathrm {BD}(\Omega )} \alpha \Vert Du-w\Vert _{\mathcal {M}}+\beta \Vert \mathcal {E}w\Vert _{\mathcal {M}}. \end{aligned}$$

Here $\alpha ,\beta$ are positive parameters and $\mathrm {BD}(\Omega )$ is the space of functions of bounded deformation, i.e. the space of all $\mathrm {L}^{1}(\Omega )$ functions *w*, whose symmetrised distributional derivative $\mathcal {E}w$ is a finite Radon measure. This is a less regular space than the usual space of functions of bounded variation $\mathrm {BV}(\Omega )$ for which the full gradient *Du* is required to be a finite Radon measure. Note that if the variable *w* in the definition (1.4) is forced to be the gradient of another function then we obtain the classical infimal convolution regulariser of Chambolle–Lions [9]. In that sense $\mathrm {TGV}$ can be seen as a particular instance of infimal convolution, optimally balancing first and second-order information.

In the discrete formulation of $\mathrm {TGV}$ (as well as for $\mathrm {TV}$) the Radon norm is interpreted as an $\mathrm {L}^{1}$ norm. The motivation for the current and the follow-up paper [8] is to explore the capabilities of $\mathrm {L}^{p}$ norms within first-order regularisation functionals designed for image processing purposes. The use of $\mathrm {L}^{p}$ norms for $p> 1$ has been exploited in different contexts—infinity and *p*-Laplacian (cf. e.g. [16] and [26] respectively).

### Our Contribution

Comparing the definition (1.1) with the definition of $\mathrm {TGV}$ in (1.4), we see that the Radon norm of the symmetrised gradient of *w* has been substituted by the $\mathrm {L}^{p}$ norm of *w*, thus reducing the order of regularisation. Up to our knowledge, this is the first paper that provides a thorough analysis of $\mathrm {TV}$–$\mathrm {L}^{p}$ infimal convolution models (1.1) in this generality. We show that the minimisation in (1.1) is well-defined and that $\mathrm {TVL}_{\alpha ,\beta }^{p}(u)<\infty$ if and only if $\mathrm {TV}(u)<\infty$. Hence $\mathrm {TVL}_{\alpha ,\beta }^{p}$ regularised images belong to $\mathrm {BV}(\Omega )$ as desired.

In order to get more insight in the regularising mechanism of the $\mathrm {TVL}_{\alpha ,\beta }^{p}$ functional we provide a detailed and rigorous analysis of its one-dimensional version of the corresponding $\mathrm {L}^{2}$ fidelity denoising problem

1.5 $$\begin{aligned} \min _{u\in \mathrm {BV}(\Omega )}\frac{1}{2}\Vert f-u\Vert _{\mathrm {L}^{2}(\Omega )}^{2}+\mathrm {TVL}_{\alpha ,\beta }^{p}(u). \end{aligned}$$

For the denoising problem (1.5) with $p=2$ we also compute exact solutions for simple one-dimensional data. We show that the obtained solutions are piecewise smooth, in contrast to $\mathrm {TV}$ (piecewise constant) and $\mathrm {TGV}$ (piecewise affine) solutions. Moreover, we show that for $p=2$, the 2-homogeneous analogue of the functional (1.1)

1.6 $$\begin{aligned} \mathrm {TVL}_{\alpha ,\beta }^{2-hom}(u)=\min _{w\in \mathrm {L}^{2}(\Omega )} \alpha \Vert Du-w\Vert _{\mathcal {M}}+\frac{\beta }{2}\Vert w\Vert _{\mathrm {L}^{2}(\Omega )}^{2}, \end{aligned}$$

is equivalent to a variant of *Huber*$\mathrm {TV}$ [24], with the functional (1.6) having a close connection to (1.1) itself. Huber total variation is a smooth approximation of total variation and even though it has been widely used in the imaging and inverse problems community, it has not been analysed adequately. Hence, as a by-product of our analysis, we compute exact solutions of the one-dimensional Huber TV denoising problem. An analogous connection of the $\mathrm {TVL}_{\alpha ,\beta }^{p}$ functional with a generalised Huber $\mathrm {TV}$ regularisation is also established for general *p*.

We proceed with exhaustive numerical experiments focusing on (1.5). Our analysis is confirmed by the fact that the analytical results coincide with the numerical ones. Furthermore, we observe that even though a first-order regularisation functional is used, we are capable of eliminating the staircasing effect, similar to Huber $\mathrm {TV}$. By using the *Bregman iteration* version of our method [32], we are also able to enhance the contrast of the reconstructed images, obtaining results very similar in quality to the $\mathrm {TGV}$ ones. We observe numerically that high values of *p* promote almost affine structures similar to second-order regularisation methods. We shed more light of this behaviour in the follow-up paper [8] where we study in depth the case $p=\infty$. Let us finally note that we also consider a modified version of the functional (1.1) where *w* is restricted to be a gradient of another function leading to the more classical infimal convolution setting. Even though, this modified model is not so successful in staircasing reduction, it is effective in decomposing an image into piecewise constant and smooth parts.

### Organisation of the Paper

After the introduction we proceed with the introduction of our model in Sect. 2. We prove the well-posedness of (1.1), we provide an equivalent definition and we prove its Lipschitz equivalence with the $\mathrm {TV}$ seminorm. We finish this section with a well-posedness result of the corresponding $\mathrm {TVL}_{\alpha ,\beta }^{p}$ regularisation problem using standard tools.

In Sect. 3 we establish a link between the $\mathrm {TVL}_{\alpha ,\beta }^{p}$ functional and its *p*-homogeneous analogue (using the *p*-th power of $\Vert \cdot \Vert _{\mathrm {L}^{p}(\Omega )}$). The *p*-homogeneous functional (for $p=2$) is further shown to be equivalent to Huber total variation, while analogous results are obtained for $p\ne 2$.

We study the corresponding one-dimensional model in Sect. 4 focusing on the $\mathrm {L}^{2}$ fidelity denoising case. More specifically, after deriving the optimality conditions using Fenchel–Rockafellar duality in Sect. 4.1, we explore the structure of solutions in Sect. 4.2. In Sect. 4.3 we compute exact solutions for the case $p=2$, considering a simple step function as data.

In Sect. 5 we present a variant of our model suitable for image decomposition purposes, i.e. geometric decomposition into piecewise constant and smooth structures.

Section 6 focuses on numerical experiments. Confirmation of the obtained one-dimensional analytical results is done in Sect. 6.2, while two-dimensional denoising experiments are performed in Sect. 6.3 using the split Bregman method. There, we show that our approach can lead to elimination of the staircasing effect and we also show that by using a Bregmanised version we can also enhance the contrast, achieving results very close to $\mathrm {TGV}$, a method considered state of the art in the context of variational regularisation. We finish the section with some image decomposition examples and we summarise our results in Sect. 7.

In the appendix, we remind the reader of some basic facts from the theory of Radon measures and $\mathrm {BV}$ functions.

## Basic Properties of the $\mathrm {TVL}_{\alpha ,\beta }^{p}$ Functional

In this section, we introduce the $\mathrm {TVL}_{\alpha ,\beta }^{p}$ functional (1.1) as well as some of its main properties. For $\alpha ,\beta >0$ and $1<p\le \infty$, we define $\mathrm {TVL}^{p}_{\alpha ,\beta }: \mathrm {L}^{1}(\Omega )\rightarrow \overline{\mathbb {R}}$ (where $\overline{\mathbb {R}}:=\mathbb {R}\cup \{+\infty \}$) as follows:While in the present paper we mainly focus on the finite *p* case, the results of this section are stated and proved for $p=\infty$ as well, since the proofs are similar.

The next proposition asserts that the minimisation in (1.1) is indeed well-defined. We omit the proof, which is based on standard coercivity and weak lower semicontinuity techniques:

### **Proposition 2.1**

Let $u\in \mathrm { BV(\Omega )}$ with $1< p\le \infty$ and $\alpha ,\beta >0$. Then the minimum in the definition (1.1) is attained.

Another useful formulation of the definition (1.1) is the *dual* formulation:2.1where *q* denotes here the conjugate exponent of *p*, see (8.4). The following proposition shows that the two expressions coincide indeed. Recall first that for a functional $F:X\rightarrow \overline{\mathbb {R}}$ the effective domain is defined as $\mathrm{dom}F = \left\{ x\in X: F(x)<\infty \right\}$, while the indicator and characteristic functions of $A\subseteq X$ are defined as

$$\begin{aligned} \mathbb {I}_{A}(x)= {\left\{ \begin{array}{ll} 0,&{} \text{ if } x\in A,\\ \infty ,&{} \text{ if } x\notin A,\\ \end{array}\right. }\quad \text{ and }\quad \mathcal {X}_{A}(x)= {\left\{ \begin{array}{ll} 1,&{} \text{ if } x\in A,\\ 0,&{} \text{ if } x\notin A,\\ \end{array}\right. } \end{aligned}$$

respectively. As usual, we denote by $\left\langle \cdot ,\cdot \right\rangle _{}$ the duality product of *X* and its dual $X^{*}$. Finally, recall that the convex conjugate $F^{*}:X^{*}\rightarrow \overline{\mathbb {R}}$ of *F* is defined as $F^{*}(x^{*})=\sup \limits _{x\in X}\left\langle x^{*},x\right\rangle _{}-F(x)$.

### **Proposition 2.2**

Let $u\in \mathrm { BV(\Omega )}$ and $1<p\le \infty$ then

### *Proof*

First notice that in (2.1), we can replace $C^1_c(\Omega )$ by $C^1_0(\Omega )$, since $\overline{C^1_c(\Omega )}=C^1_0(\Omega )$ with the closure taken with respect to the $C^{1}$ norm, i.e. . We defineThen, we can rewrite (2.1) asWe can establish the following relation

$$\begin{aligned} \inf _{\phi \in X} \left\{ F_{1}(\phi )+F_{2}(\phi )\right\} +\min _{w\in X^{*}}\left\{ F_{1}^{*}(w)+F_{2}^{*}(w)\right\} =0, \end{aligned}$$

i.e. the absence of duality gap between the primal and the dual problems [15, Chapter III], provided that the set

$$\begin{aligned} \bigcup _{\lambda \ge 0}\lambda \left( \mathrm{dom}F_{2} - \mathrm{dom}F_{1}\right) , \end{aligned}$$

is a closed vector space [2]. This is indeed true since on one hand we have

$$\begin{aligned} \bigcup _{\lambda \ge 0}\lambda \left( \mathrm{dom}F_{2} - \mathrm{dom}F_{1}\right) \subset X, \end{aligned}$$

and on the other hand, for every $\phi \in X$, we can write $\phi =\lambda (\lambda ^{-1}\phi -0)$ with  and $0\in \mathrm{dom}F_{1}$. Since $u\in \mathrm {BV}(\Omega )$, then $\mathrm {TVL}^{p}_{\alpha ,\beta }$ is finite, see also Proposition 2.4. Hence, $F_{1}^{*}(w)<\infty$, $F_{2}^{*}(w)<\infty$ andwhere we have used the fact that, with a density argument, the function $w: C_{0}^{1}\rightarrow \mathbb {R}$ can be extended in the whole $\mathrm {L}^{q}(\Omega )$ as a bounded, linear functional and using the Riesz representation theorem, we deduce that it is actually an $\mathrm {L}^{p}$ function. Similarly, we haveThus the desired equality is proven.

### *Remark 2.3*

Note that using the dual formulation of $\mathrm {TVL}^{p}_{\alpha ,\beta }$ one can easily derive that the functional is lower semicontinuous with respect to the strong $\mathrm {L}^{1}$ topology since it is a pointwise supremum of continuous functions.

The following lemma shows that the $\mathrm {TVL}^{p}_{\alpha ,\beta }$ functional is Lipschitz equivalent to the total variation.

### **Proposition 2.4**

Let $u\in \mathrm {L^{1}(\Omega )}$ and $1<p\le \infty$. Then $\mathrm {TVL}^{p}_{\alpha ,\beta }(u)<\infty$ if and only if $u\in \mathrm {BV}(\Omega )$. Moreover there exist constants $C_{1}=\alpha$ and $C_{2}=(C\tilde{C})^{-1}$, where $C=\max \big (1,|\Omega |^{\frac{1}{q}}\big )$ and $\tilde{C}=\max \big (\frac{1}{\alpha },\frac{1}{\beta }\big )$ such that2.2Finally in the special case where

2.3 $$\begin{aligned} \frac{\beta }{\alpha }\ge |\Omega |^{\frac{1}{q}}, \end{aligned}$$

then2.4

### *Proof*

Let $u\in \mathrm {BV}(\Omega )$. Using the definition (1.1) we have thatfor every $w\in \mathrm {L}^{p}(\Omega )$. Setting $w=0$ and $C_{1}=\alpha$, we obtain2.5For the other direction, for any $w\in \mathrm {L}^{p}(\Omega )\subset \mathrm {L^{1}(\Omega )}$, by the triangle inequality we get2.6with $C=\max \big (1,|\Omega |^{\frac{1}{q}} \big )$. By setting $C_{2}$ as in the statement of the lemma we obtainwhich, by minimising over *w*, yields the left-hand side inequality in (2.2).

Finally, observe simply that if (2.3) holds then from (2.6) we getand thus minimising again over *w* and combining (2.5) we get (2.4).

Notice that when (2.3) holds then the above proposition implies that $w=0$ is an admissible solution to the definition of $\mathrm {TVL}_{\alpha ,\beta }^{p}(u)$, i.e.

$$\begin{aligned}&0\in \underset{w\in \mathrm {L}^{p}(\Omega )}{{\text {argmin}}}\; \alpha \Vert Du-w\Vert _{\mathcal {M}}+\beta \Vert w\Vert _{\mathrm {L}^{p}(\Omega )},\\&\quad \text {for all }u\in \mathrm {BV}(\Omega ). \end{aligned}$$

However, in general we cannot prove that this solution is unique.

Having shown the basic properties of the $\mathrm {TVL}^{p}_{\alpha ,\beta }$ functional, we can use it as a regulariser for variational imaging problems of the type

2.7 $$\begin{aligned} \min _{u\in \mathrm {L}^{s}(\Omega )\cap \mathrm {BV}(\Omega )} \frac{1}{s} \Vert f-Tu\Vert _{\mathrm {L}^{s}(\Omega )}^{s}+\mathrm {TVL}^{p}_{\alpha ,\beta }(u),\quad s\ge 1,\nonumber \\ \end{aligned}$$

where $T: \mathrm {L}^{s}(\Omega )\rightarrow \mathrm {L}^{s}(\Omega )$ is a bounded, linear operator and $f\in \mathrm {L}^{s}(\Omega )$. We conclude our analysis with existence and uniqueness results for the minimisation problem (2.7).

### **Theorem 2.5**

Let $1<p\le \infty$ and $f\in \mathrm {L}^{s}(\Omega )$. If $T(\mathcal {X}_{\Omega })\ne 0$ then there exists a solution $u\in \mathrm {L}^{s}(\Omega )\cap \mathrm {BV}(\Omega )$ for the problem (2.7). If $s>1$ and *T* is injective then the solution is unique.

### *Proof*

The proof is a straightforward application of the direct method of calculus of variations. We simply take advantage of the inequality (2.2) and the compactness theorem in $\mathrm {BV}(\Omega )$, see Appendix, along with the lower semicontinuity property of $\mathrm {TVL}^{p}_{\alpha ,\beta }$. We also refer the reader to the corresponding proofs in [34, 39].$\square$

Since we are mainly interested in studying the regularising properties of $\mathrm {TVL}^{p}_{\alpha ,\beta }$, in what follows we focus on the case where $s=2$ and *T* is the identity function (denoising task) where rigorous analysis can be carried out. From now on, we also focus on the case where *p* is finite, as the case $p=\infty$ is studied in the follow-up paper [8]. We thus define the following problemor equivalently 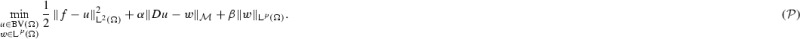


## The *p*-Homogeneous Analogue and Relation to Huber TV

Before we proceed to a detailed analysis of the one-dimensional version of $(\mathcal {P})$, in this section we consider its *p*-homogeneous analogue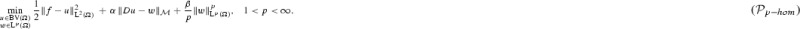 We show in Proposition 3.2 that there is a strong connection between the models $(\mathcal {P})$ and $(\mathcal {P}_{p-hom})$. The reason for the introduction of $(\mathcal {P}_{p-hom})$ is that, in certain cases, it is technically easier to derive exact solutions for $(\mathcal {P}_{p-hom})$ rather than for $(\mathcal {P})$ straightforwardly, see Sect. 4.3. Moreover, we can guarantee the uniqueness of the optimal $w^{*}$ in $(\mathcal {P}_{p-hom})$, sinceand thus $w^{*}$ is unique as a minimiser of a strictly convex functional. The next proposition states that, unless *f* is a constant function then the optimal $w^{*}$ in $(\mathcal {P}_{p-hom})$ cannot be zero but nonetheless converges to zero as $\beta \rightarrow \infty$. In essence, this means that one cannot obtain $\mathrm {TV}$ type solutions with the *p*-homogeneous model.

### **Proposition 3.1**

Let $1<p<\infty$, $f\in \mathrm {L}^{2}(\Omega )$ and let $(w^{*},u^{*})$ be an optimal solution pair of the *p*-homogeneous problem $(\mathcal {P}_{p-hom})$. Then $w^{*}=0$ if and only if *f* is a constant function. For general data *f*, we have that $w^{*}\rightarrow 0$ in $\mathrm {L}^{p}(\Omega )$ when $\beta \rightarrow \infty$.

### *Proof*

It follows immediately that if *f* is constant then (0, *f*) is the optimal pair for $(\mathcal {P}_{p-hom})$. Suppose that $(w^{*},u^{*})$ solve $(\mathcal {P}_{p-hom})$. Notice that in this case we also have

3.1 $$\begin{aligned} u^{*}&= \underset{u\in \mathrm {BV}(\Omega )}{{\text {argmin}}} \; \frac{1}{2} \Vert f-u\Vert _{\mathrm {L}^{2}(\Omega )}^{2}+\alpha \Vert Du-w^{*}\Vert _{\mathcal {M}}. \end{aligned}$$

Suppose now that $w^{*}=0$. Then (3.1) becomes

3.2 $$\begin{aligned} u^{*} = \underset{u\in \mathrm {BV}(\Omega )}{{\text {argmin}}} \; \frac{1}{2} \Vert f-u\Vert _{\mathrm {L}^{2}(\Omega )}^{2}+\alpha \Vert Du\Vert _{\mathcal {M}}. \end{aligned}$$

Furthermore, since $(0,u^{*})$ solve $(\mathcal {P}_{p-hom})$, then for every $u\in C_{c}^{\infty }(\Omega )$ and $\epsilon >0$, the pair $(\epsilon \nabla u,u^{*}+\epsilon u )$ is suboptimal for $(\mathcal {P}_{p-hom})$, i.e.

$$\begin{aligned}&\frac{1}{2}\Vert f-u^{*}\Vert _{\mathrm {L}^{2}(\Omega )}^{2}+\alpha \Vert Du^{*}\Vert _{\mathcal {M}}\le \frac{1}{2}\Vert (f-u^{*})-\epsilon u\Vert _{\mathrm {L}^{2}(\Omega )}^{2}\\&\quad +\alpha \Vert D(u^{*}+\epsilon u)-\epsilon \nabla u\Vert _{\mathcal {M}}+\frac{\beta }{p}\Vert \epsilon \nabla u\Vert _{\mathrm {L}^{p}(\Omega )}^{p}, \end{aligned}$$

from which we take

$$\begin{aligned} \frac{1}{2}\Vert f-u^{*}\Vert _{\mathrm {L}^{2}(\Omega )}^{2}&\le \frac{1}{2}\Vert (f-u^{*})-\epsilon u\Vert _{\mathrm {L}^{2}(\Omega )}^{2}\\&\quad +\frac{\beta }{p}\Vert \epsilon \nabla u\Vert _{\mathrm {L}^{p}(\Omega )}^{p}\Leftrightarrow \\ 0&\le \frac{\epsilon ^{2}}{2}\Vert u\Vert _{\mathrm {L}^{2}(\Omega )}^{2}-\epsilon \int _{\Omega }(f-u^{*})u\,\mathrm{d}x\\&\quad +\frac{\beta \epsilon ^{p}}{p}\Vert \nabla u\Vert _{\mathrm {L}^{p}(\Omega )}^{p}. \end{aligned}$$

By dividing the last inequality by $\epsilon$ and taking the limit $\epsilon \rightarrow 0$ we have that $\int _{\Omega }(f-u^{*})u\,\mathrm{d}x\le 0$. By considering the analogous perturbations $u^{*}-\epsilon u$ , we obtain similarly that $\int _{\Omega }(f-u^{*})u\,\mathrm{d}x\ge 0$ and thus

$$\begin{aligned} \int _{\Omega }(f-u^{*})u\,\mathrm{d}x= 0,\quad \forall u\in C_{c}^{\infty }(\Omega ). \end{aligned}$$

Hence $u^{*}=f$ and by taking the optimality condition of (3.2) we get that , which implies that $Df=0$, i.e. *f* is a constant function.

For the last part of the proposition, (supposing $f\ne 0$), simply observe that for every $u\in \mathrm {BV}(\Omega )$ and $w\in \mathrm {L}^{p}(\Omega )$ we have that

$$\begin{aligned}&\frac{1}{2}\Vert f-u^{*}\Vert _{\mathrm {L}^{2}(\Omega )}^{2}+\alpha \Vert Du^{*}-w^{*}\Vert _{\mathcal {M}}+\frac{\beta }{p} \Vert w^{*}\Vert _{\mathrm {L}^{p}(\Omega )}^{p} \\&\qquad \le \frac{1}{2}\Vert f-u\Vert _{\mathrm {L}^{2}(\Omega )}^{2}+\alpha \Vert Du-w\Vert _{\mathcal {M}}+\frac{\beta }{p} \Vert w\Vert _{\mathrm {L}^{p}(\Omega )}^{p}, \end{aligned}$$

and by setting $u=w=0$, we obtain

$$\begin{aligned} \frac{1}{p} \Vert w^{*}\Vert _{\mathrm {L}^{p}(\Omega )}^{p}\le \frac{1}{2\beta } \Vert f\Vert _{\mathrm {L}^{2}(\Omega )}^{2}, \end{aligned}$$

and thus $\Vert w^{*}\Vert _{\mathrm {L}^{p}(\Omega )}^{p}\rightarrow 0$ when $\beta \rightarrow \infty$.$\square$

We further establish a connection between the 1-homogeneous $(\mathcal {P})$ and the *p*-homogeneous model $(\mathcal {P}_{p-hom})$:

### **Proposition 3.2**

Let $1<p<\infty$ and $f\in \mathrm {L}^{2}(\Omega )$ not a constant. A pair $(w^{*},u^{*})$ is a solution of $(\mathcal {P}_{p-hom})$ with parameters $(\alpha ,\beta _{p-hom})$ if and only if it is also a solution of $(\mathcal {P})$ with parameters $(\alpha ,\beta _{1-hom})$ where $\beta _{1-hom}=\beta _{p-hom}\Vert w^{*}\Vert _{\mathrm {L}^{p}(\Omega )}^{p-1}$.

### *Proof*

Since *f* is not a constant by the previous proposition we have that $w^{*}\ne 0$. Note that for an arbitrary function $u\in \mathrm {BV}(\Omega )$:This means that $w^{*}$ is an admissible solution for both problems $(\mathcal {P})$ and $(\mathcal {P}_{p-hom})$, with the corresponding set of parameters $(\alpha ,\beta _{1-hom})$ and $(\alpha ,\beta _{p-hom})$, respectively. The fact that the same holds for $u^{*}$ as well, comes from the fact that in both problems we have

$$\begin{aligned} u^{*}\in \underset{u\in \mathrm {BV}(\Omega )}{{\text {argmin}}} \frac{1}{2}\Vert f-u\Vert _{\mathrm {L}^{2}(\Omega )}^{2}+\alpha \Vert Du-w^{*}\Vert _{\mathcal {M}}. \end{aligned}$$

Finally, it turns out that for $$p=2$$, problem $$(\mathcal {P}_{p-hom})$$ is essentially equivalent to the widely used Huber total variation regularisation, [24]. In fact we can show that for $$1<p<\infty $$$$(\mathcal {P}_{p-hom})$$ is equivalent to a generalised Huber total variation regularisation, see also [23]. This is proved in the next proposition.

### **Proposition 3.3**

Let $1<p<\infty$ and consider the functional $\mathrm {TVL}_{\alpha ,\beta }^{p-hom}:\mathrm {BV}(\Omega )\rightarrow \mathbb {R}$ with

3.3 $$\begin{aligned} \mathrm {TVL}_{\alpha ,\beta }^{p-hom}(u)=\min _{w\in \mathrm {L}^{p}(\Omega )} \alpha \Vert Du-w\Vert _{\mathcal {M}}+\frac{\beta }{p}\Vert w\Vert _{\mathrm {L}^{p}(\Omega )}^{p}. \end{aligned}$$

Then

$$\begin{aligned} \mathrm {TVL}_{\alpha ,\beta }^{p-hom}(u)=\int _{\Omega }\varphi _{p}(\nabla u)\,\mathrm{d}x + \alpha |D^{s}u|(\Omega ), \end{aligned}$$

where $$\varphi _{p}:\mathbb {R}^{d}\rightarrow \mathbb {R}$$ with

$$\begin{aligned} \varphi _{p}(x)= {\left\{ \begin{array}{ll} \alpha |x|-\left( 1-\frac{1}{p}\right) \frac{\alpha }{\lambda ^{\frac{1}{p-1}}}, &{} |x|\ge \frac{1}{\lambda ^{\frac{1}{p-1}}},\\ \frac{\beta }{p} |x|^{p}, &{} |x|\le \frac{1}{\lambda ^{\frac{1}{p-1}}}, \end{array}\right. }\qquad \text {where}\quad \lambda :=\frac{\beta }{\alpha }, \end{aligned}$$

and $D^{s}u$ denotes the singular part of the measure *Du*, cf. Appendix.

### *Proof*

We have

$$\begin{aligned} \mathrm {TVL}_{\alpha ,\beta }^{p-hom}(u)&= \min _{w\in \mathrm {L}^{p}(\Omega )} \alpha \Vert Du-w\Vert _{\mathcal {M}}+\frac{\beta }{p}\Vert w\Vert _{\mathrm {L}^{p}(\Omega )}^{p}\\&= \alpha |D^{s}u|(\Omega )+\alpha \min _{w\in \mathrm {L}^{p}(\Omega )} \int _{\Omega } |\nabla u-w|\\&\quad \quad +\frac{\beta }{p\alpha }|w|^{p}\,\mathrm{d}x. \end{aligned}$$

Thus we can focus on the minimisation problem

3.4 $$\begin{aligned} \min _{w\in \mathrm {L}^{p}(\Omega )} \int _{\Omega } |\nabla u-w|+\frac{\beta }{p\alpha }|w|^{p}\,\mathrm{d}x. \end{aligned}$$

Baring in mind that (as it can easily checked) for $c\in \mathbb {R}^{d}$ and $\lambda >0$,

$$\begin{aligned} \underset{y\in \mathbb {R}^{d}}{{\text {argmin}}}\; |c-y|+\frac{\lambda }{p}|y|^{p}= {\left\{ \begin{array}{ll} \frac{1}{\lambda ^{\frac{1}{p-1}}}\frac{c}{|c|} &{} \hbox { if } |c|\ge \frac{1}{\lambda ^{\frac{1}{p-1}}},\\ c &{} \hbox { if } |c|< \frac{1}{\lambda ^{\frac{1}{p-1}}}, \end{array}\right. } \end{aligned}$$

and

$$\begin{aligned} \min _{y\in \mathbb {R}^{d}} |c-y|+\frac{\lambda }{p}|y|^{p}= {\left\{ \begin{array}{ll} |c|- \left( 1-\frac{1}{p} \right) \frac{1}{\lambda ^{\frac{1}{p-1}}} &{} \text{ if } |c|\ge \frac{1}{\lambda ^{\frac{1}{p-1}}},\\ \frac{\lambda }{p}|c|^{p} &{} \text{ if } |c|< \frac{1}{\lambda ^{\frac{1}{p-1}}}, \end{array}\right. } \end{aligned}$$

it is straightforwardly verified setting $\lambda =\beta /\alpha$ that the function

$$\begin{aligned} w^{*}=\lambda ^{-\frac{1}{p-1}}\frac{\nabla u}{|\nabla u|}\mathcal {X}_{\left\{ |\nabla u|\ge \lambda ^{-\frac{1}{p-1}}\right\} }+\nabla u \mathcal {X}_{\left\{ |\nabla u|< \lambda ^{-\frac{1}{p-1}}\right\} }, \end{aligned}$$

belongs to $\mathrm {L}^{\infty }(\Omega )\subset \mathrm {L}^{p}(\Omega )$ and solves (3.4) with optimal value equal to $\frac{1}{\alpha }\int _{\Omega }\varphi _{p}(\nabla u)\,\mathrm{d}x$.$\square$

Note that in the special case $p=2$ we recover the classical Huber total variation regularisation since

$$\begin{aligned} \varphi _{2}(x)= {\left\{ \begin{array}{ll} \alpha |x|-\frac{\alpha ^{2}}{2\beta } &{} |x|\ge \frac{\alpha }{\beta },\\ \frac{\beta }{2} |x|^{2}, &{} |x|\le \frac{\alpha }{\beta }, \end{array}\right. } \end{aligned}$$

i.e. in that case we have quadratic penalisation for small gradients (*p*–power penalisation for the general $1<p<\infty$) and linear penalisation for large gradients.

**Fig. 1:**
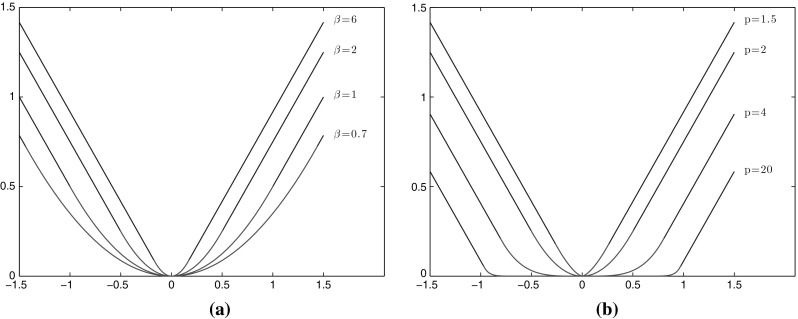
Illustration of the forms of the Huber-type functions $\varphi _{p}$ of Proposition 3.3. Their linear and *p*–power parts are plotted with *blue* and *red* colour, respectively. **a** Huber functions $\varphi _{2}$ with fixed $p=2$, $\alpha =1$ and varying $\beta$. **b** Generalised Huber functions $\varphi _{p}$ with fixed $\alpha =1$, $\beta =2$ and varying *p* (Color figure online)

For the reader’s convenience, in Fig. 1 we have plotted some of the functions $\varphi _{p}$ in order to illustrate how their form changes when their parameters vary. Note for instance in Fig. 1a how $\phi _{2}$ is converging to an absolute type function when $\beta$ is getting large, i.e. approaching a total variation regularisation. This can also be seen from Proposition 3.1 where the optimal variable *w* is converging to 0 when $\beta \rightarrow \infty$. On the other hand when *p* is getting large, Fig. 1b, small gradients are essentially not penalised at all, allowing the gradient to be almost constant, equal to its maximum value, leading to piecewise affine structures. We refer to some of the numerical examples in Sect. 6.2 and also the second part of this paper [8] where the case $p=\infty$ is examined in detail.

## The One-Dimensional Case

In order to get more insights into the structure of solutions of the problem $(\mathcal {P})$, in this section we study its one-dimensional version. As above, we focus on the finite *p* case, i.e. $1<p<\infty$. The case $p=\infty$ leads to several additional complications and will be subject of a forthcoming paper [8]. For this section $\Omega \subset \mathbb {R}$ is an open and bounded interval, i.e. $\Omega =(a,b)$. Our analysis follows closely the ones in [6] and [33] where the one dimensional $\mathrm {L}^{1}$–$\mathrm {TGV}$ and $\mathrm {L}^{2}$–$\mathrm {TGV}$ problems are studied, respectively.

### Optimality Conditions

In this section, we derive the optimality conditions for the one-dimensional problem $(\mathcal {P})$. We initially start our analysis by defining the predual problem $(\mathcal {P}^{*})$, proving existence and uniqueness for its solutions. We employ again the Fenchel–Rockafellar duality theory in order to find a connection between the solutions of the predual and primal problems.

We define the predual problem $(\mathcal {P}^{*})$ as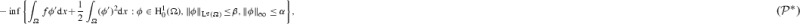 where as always *q* is the conjugate exponent of *p*. Existence and uniqueness for the solutions of $(\mathcal {P}^{*})$ can be verified by standard arguments:

#### **Proposition 4.1**

For $f \in \mathrm {L}^2(\Omega )$, the predual problem $(\mathcal {P}^{*})$ admits a unique solution in $\mathrm {H}_{0}^{1}(\Omega ).$

The next proposition justifies the term predual for the problem $(\mathcal {P}^{*})$.

#### **Proposition 4.2**

The dual problem of $(\mathcal {P}^{*})$ is equivalent to the problem $(\mathcal {P})$ in the sense that (*w*, *u*) is a solution of the dual of $(\mathcal {P}^{*})$ if and only if $(w,u)\in \mathrm {L}^{p}(\Omega )\times \mathrm {BV}(\Omega )$ and solves $(\mathcal {P})$.

#### *Proof*

Observe that we can also write down the predual problem $(\mathcal {P}^{*})$ using the following equivalent formulation:

4.1 $$\begin{aligned} -\inf _{(\phi ,\xi )\in X}F_{1}(\phi ,\xi ) + F_{2}(K(\phi ,\xi )), \end{aligned}$$

where $X=\mathrm {H_{0}^{1}(\Omega )}\times \mathrm {H_{0}^{1}(\Omega )}$, $Y=\mathrm {H_{0}^{1}(\Omega )}\times \mathrm {L^{2}(\Omega )}$ and4.2We denote the infimum in $(\mathcal {P}^{*})$ by $\inf \mathcal {P}^{*}$. Then, it is immediate that

$$\begin{aligned} -\inf \mathcal {P}^{*}=-\inf _{(\phi ,\xi )\in X}F_{1}(\phi ,\xi ) + F_{2}(K(\phi ,\xi )). \end{aligned}$$

The dual problem of (4.1), see [15], is defined as

4.3 $$\begin{aligned} \min _{(w,u)\in Y^{*}} F_{1}^{*}(- K^{\star }(w,u))+F_{2}^{*}(w,u), \end{aligned}$$

where $K^{\star }$ here denotes the adjoint of *K*. Let $(\sigma ,\tau )$ be elements of $X^{*}=\mathrm {H}_{0}^{1}(\Omega )^{*}\times \mathrm {H}_{0}^{1}(\Omega )^{*}$. The convex conjugate of $F_{1}$ can be written as4.4By standard density arguments and using the Riesz representation theorem we have4.5Moreover, we have

$$\begin{aligned}&\left\langle -K^{\star }(w,u),(\phi ,\xi )\right\rangle _{}\\&\quad =-\left\langle (w,u),K(\phi ,\xi )\right\rangle _{} =-\left\langle (w,u),(\xi -\phi ,\xi ')\right\rangle _{}\\&\quad = -\left\langle w,\xi \right\rangle _{}+\left\langle w,\phi \right\rangle _{}-\left\langle u,\xi '\right\rangle _{} = \left\langle Du-w,\xi \right\rangle _{}+\left\langle w,\phi \right\rangle _{}. \end{aligned}$$

Since $F_{1}^{*}(-K^{\star }(w,u))<\infty$, $F_{2}^{*}(w,u)<\infty$, we obtain that4.6and4.7$\square$

We next verify that we have no duality gap between the two minimisation problems $(\mathcal {P})$ and $(\mathcal {P}^{*})$. The proof of the following proposition follows the proof of the corresponding proposition in [6]. We slightly modify it for our case.

#### **Proposition 4.3**

Let $F_{1}, F_{2}, K, X, Y$ be defined as in (4.2). Then

4.8 $$\begin{aligned} \bigcup _{\lambda \ge 0}\lambda (\mathrm{dom}F_{2} - K (\mathrm{dom}F_{1}))=Y \end{aligned}$$

and hence it is a closed vector space. Thus [2]

4.9 $$\begin{aligned}&\min _{(\phi ,\xi )\in X}F_{1}(\phi ,\xi )+F_{2}(K(\phi ,\xi )) \nonumber \\&\quad +\min _{(w,u)\in Y^{*}} F_{1}^{*}(- K^{\star }(w,u))+F_{2}^{*}(w,u)=0. \end{aligned}$$

#### *Proof*

Let $(v,\psi )\in Y$ and define $\psi _{0}(x)=c_{1}$, where

4.10 $$\begin{aligned} c_{1}=\frac{1}{|\Omega |}\int _{\Omega }\psi (x)\, \mathrm{d}x. \end{aligned}$$

Now let $\xi (x)=\int _{a}^{x}(\psi _{0}-\psi )(y) \,dy$. Since by construction, $\xi '=\psi _{0}-\psi \in \mathrm {L^{2}(\Omega )}$ with $\xi (a)=\xi (b)=0$, we have that $\xi \in \mathrm {H}_{0}^{1}(\Omega )$. Furthermore, let $\phi = - v+\xi \in \mathrm {H}_{0}^{1}(\Omega )$ and $(\phi ,\xi )\in X$ with

$$\begin{aligned} (v,\psi )= & {} (\xi -\phi ,\psi _{0}-\xi ')=(0,\psi _{0})-(\xi -\phi ,\xi ')\\= & {} (0,\psi _{0})-K(\phi ,\xi ). \end{aligned}$$

Choosing appropriately $\lambda >0$ such that , , we can write

$$\begin{aligned} (v,\psi )=\lambda \left( (0,\lambda ^{-1}\psi _{0})-K(\lambda ^{-1}\phi ,\lambda ^{-1}\xi )\right) , \end{aligned}$$

with $\mathrm{dom}F_{2} = \{0\} \times \mathrm {L^{2}(\Omega )}$ and . Since $(v,\psi )\in Y$ were chosen arbitrarily, (4.8) holds.$\square$

Since there is no duality gap, we can find a relationship between the solutions of $(\mathcal {P}^{*})$ and $(\mathcal {P})$ via the following optimality conditions.

#### **Theorem 4.4**

(Optimality conditions) Let $1<p\le \infty$ and $f\in \mathrm {L}^{2}(\Omega )$. A pair $(w,u)\in \mathrm {L}^{p}(\Omega ) \times \mathrm {BV}(\Omega )$ is a solution of $(\mathcal {P})$ if and only if there exists a function $\phi \in \mathrm {H_{0}^{1}(\Omega )}$ such that

4.11 $$\begin{aligned} \begin{aligned}&\phi ' = u-f,\\&\phi \in \alpha \mathrm{Sgn}(Du-w),\\ \end{aligned} \end{aligned}$$

and

4.12 $$\begin{aligned} \begin{aligned} {\left\{ \begin{array}{ll} \phi \in \{ \tilde{\phi }\in \mathrm {L}^{q}(\Omega ) : \Vert \tilde{\phi }\Vert _{\mathrm {L}^{q}(\Omega )} \le \beta \} &{} \text{ if } w=0,\\ \phi =\beta \frac{|w|^{p-2}w}{ \Vert w \Vert _{\mathrm {L}^{p}(\Omega )}^{(p-1)}} &{} \text{ if } w\ne 0. \end{array}\right. } \end{aligned} \end{aligned}$$

#### *Proof*

Since there is no duality gap, the optimality conditions read [15, Prop. 4.1(III)]:

4.13 $$\begin{aligned} (\phi ,\xi )&\in \partial F_{1}^{*}(-K^{\star }(w,u)),\end{aligned}$$

4.14 $$\begin{aligned} K(\phi ,\xi )&\in \partial F_{2}^{*} (w,u), \end{aligned}$$

for every $(\phi ,\xi )$ and (*w*, *u*) that solve $(\mathcal {P}^{*})$ and $(\mathcal {P})$, respectively. Hence, for every $(\sigma ,\tau )\in X^{*}$, we have the following:4.15Since $\xi \in \mathrm {H_{0}^{1}(\Omega )}\subset \mathrm {C}_{0}(\Omega )$ in one dimension, we can make use of the fact that

$$\begin{aligned} \partial \Vert \cdot \Vert _{\mathcal {M}}(Du-w)\cap C_{0}(\Omega )=\mathrm {Sgn}(Du-w)\cap C_{0}(\Omega ), \end{aligned}$$

see (8.1), and write the expressions in (4.15) as

4.16 $$\begin{aligned} \xi \in \alpha \mathrm{Sgn}(Du-w), \end{aligned}$$

and4.17Indeed, the $\mathrm {L}^{p}$ norm is an one-homogeneous functional and thus its subdifferential readsNote that for $w=0$, the above expression reduces to  for all $\sigma \in \mathrm {L}^{p}(\Omega )$, which is valid for any $z\in \mathrm {L}^{q}(\Omega )$ with , i.e. the unit ball of $\mathrm {L}^{q}(\Omega )$. If $w\ne 0$ then the subdifferential is simply the Gâteaux derivative of the $\mathrm {L}^{p}$ norm, i.e. . Finally, from (4.14) we have for every $(\hat{w},\hat{u})\in Y^{*}$Combining all the above results, we obtain the optimality conditions (4.11) and (4.12).

#### *Remark 4.5*

We observe that if $w=0$ then the conditions (4.11) coincide with the optimality conditions for the $\mathrm {L}^{2}$–$\mathrm {TV}$ minimisation problem (ROF) with parameter $\alpha$, i.e.4.18see also [37]. On the other hand when $w\ne 0$, the additional condition (4.12) depends on the value of *p* and as we will see later it allows a certain degree of smoothness in the final solution *u*.

### Structure of the Solutions

The optimality conditions (4.11) and (4.12) can help us explore the structure of the solutions for the problem $(\mathcal {P})$ and how this structure is determined by the regularising parameters $\alpha , \beta$ and the value of *p*.

We initially discuss the cases where the solution *u* of $(\mathcal {P})$ is a solution of a corresponding ROF minimisation problem i.e. $w=0$. Note that the following proposition holds for $p=\infty$ as well.

#### **Proposition 4.6**

(ROF solutions) Let *q* be the conjugate exponent of $p\in (1,\infty ]$ as defined in (8.4). If

$$\begin{aligned} \frac{\beta }{\alpha }\ge |\Omega |^{\frac{1}{q}}, \end{aligned}$$

then (0, *u*) is a solution pair for $(\mathcal {P})$ where *u* solves the ROF minimisation problem (4.18).

#### *Proof*

The proof follows immediately from Proposition 2.4.

Proposition 4.6 is valid for any dimension $d\ge 1$. It provides a rough threshold for obtaining ROF type solutions in terms of the regularising parameters $\alpha ,\beta$ and the image domain $\Omega$. However, the condition is not sharp in general since as we will see in the following sections we can obtain a sharper estimate for specific data *f*.

The following proposition in the spirit of [6, 33] gives more insight into the structure of solutions of $(\mathcal {P})$.

#### **Proposition 4.7**

Let $f\in \mathrm {BV(\Omega )}$ and suppose that $(w,u)\in \mathrm {L}^{p}(\Omega )\times \mathrm {BV}(\Omega )$ is a solution pair for $(\mathcal {P})$ with $p\in (1,\infty ]$. Suppose that $u>f$ (or $u<f$) on an open interval $I\subset \Omega$ then $(Du-w)\lfloor I = 0$, i.e. $u'=w$ on *I* and $|D^{s}u|(I)=0$.

The above proposition is formulated rigorously via the use of *good representatives* of $\mathrm {BV}$ functions, see [1], but for the sake of simplicity we rather not get into the details here. Instead we refer the reader to [6, 33] where the analogue propositions are shown for the $\mathrm {TGV}$ regularised solutions and whose proofs are similar to the one of Proposition 4.7.

We now examine the case where the solution is constant in $\Omega$, which in fact coincides with the mean value $\tilde{f}$ of the data *f*:4.19

#### **Proposition 4.8**

(Mean value solution) If the following conditions hold

4.20 $$\begin{aligned} \begin{aligned}&\alpha \ge \Vert f-\tilde{f}\Vert _{\mathrm {L}^1(\Omega )},\\&\beta \ge |\Omega |^{\frac{1}{q}}\Vert f-\tilde{f}\Vert _{\mathrm {L}^1(\Omega )}, \end{aligned} \end{aligned}$$

then the solution of $(\mathcal {P})$ is constant and equal to $\tilde{f}$.

#### *Proof*

Clearly, if *u* is a constant solution of $(\mathcal {P})$, then $Du=0$ and from inequality (2.2) we get $\mathrm {TVL}_{\alpha ,\beta }^{p}(u)=0$. Hence, we have $u=\tilde{f}$.

In general, in order to have $u=\tilde{f}$, from the optimality conditions (4.11) and (4.12), it suffices to find a function $\phi \in \mathrm {H}^{1}_{0}(\Omega )$ such thatLetting $\phi (x)=\int _{a}^{x} (f(s)-\tilde{f}) \,ds$, then obviously $\phi \in \mathrm {H}^{1}_{0}(\Omega )$ since $\phi (a)=\phi (b)=0$ and

$$\begin{aligned} |\phi (x)| \le \int _{a}^{x} |f(s)-\tilde{f}| \,ds \le \Vert f-\tilde{f}\Vert _{\mathrm {L}^1(\Omega )}<\infty . \end{aligned}$$

Therefore, . Also, since $\mathrm {L}^{\infty }(\Omega )\subset \mathrm {L}^{q}(\Omega )$ we obtainHence, it suffices to choose $\alpha$ and $\beta$ as in (4.20).

**Fig. 2:**
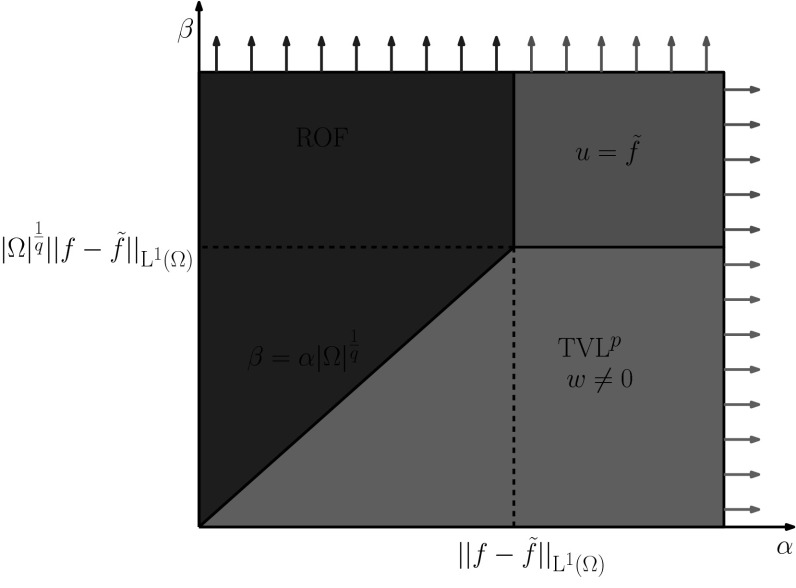
Characterisation of solutions of $(\mathcal {P})$ for any data *f*: The *blue/red* areas correspond to the ROF type solutions ($w=0$) and the *purple* area corresponds to the $\mathrm {TVL}^{p}$ solutions ($w\ne 0)$ for $1<p<\infty$. We note that the *blue/red* and *purple* areas are potentially larger or smaller, respectively as the conditions we have derived are not sharp (Color figure online)

In Fig. 2, we summarise our results so far. There, we have partitioned the set $\{\alpha >0,\beta >0\}$ into different areas that correspond to different types of solutions of the problem $(\mathcal {P})$. The red area, arising from thresholds (4.20) corresponds to the choices of $\alpha$ and $\beta$ that produce constant solutions while the blue area corresponds to ROF type solutions, according to threshold (2.3). Therefore, we can determine the area where the non-trivial solutions are obtained, i.e. $w\ne 0$, see purple region. Note that since the conditions (2.3) and (4.20) are not sharp, the blue/red and the purple areas are potentially larger or smaller, respectively than it is shown in Fig. 2.

Propositions 4.6 and 4.8 tell us how the solutions *u* behave when $\beta /\alpha$ or one of the parameters $\alpha$ and $\beta$ is large. The other limiting case is also of interest, i.e. when the parameters are small. The analogous questions have been examined in [35] for the $\mathrm {TGV}$ case in arbitrary dimension. There it is shown that whenever $\beta \rightarrow 0$ while keeping $\alpha$ fixed or $\alpha \rightarrow 0$ while keeping $\beta$ fixed, the corresponding $\mathrm {TGV}$ solutions converge to the data *f* strongly in $\mathrm {L}^{2}$. The same result holds for the $\mathrm {TVL}^{p}$ regularisation. The proof from [35] can be straightforwardly adapted to our case.

The following proposition reveals more information about the structure of solutions in the case $w \ne 0$.

#### **Proposition 4.9**

($\mathrm {TVL}^p$ solutions) Let $f\in \mathrm {BV(\Omega )}$ and suppose that $(w,u)\in \mathrm {L}^{p}(\Omega )\times \mathrm {BV}(\Omega )$ is a solution pair for $(\mathcal {P})$ with $p\in (1,\infty )$ and $w\ne 0$. Suppose that $u>f$ (or $u<f$) on an open interval $I\subset \Omega$ then the solution *u* of $(\mathcal {P})$ is obtained by4.21

#### *Proof*

Since $1<p<\infty$, $w\ne 0$ using Proposition 4.7 and the second optimality condition of (4.12), we have thatHence, using (4.11) we obtain (4.21) where .$\square$

Let us make a few remarks regarding equation (4.21) which is in fact the *p*-Laplace equation. One cannot write down a priori the boundary conditions associated with this equation on an interval *I* where $u>f$ (or $u<f$) as it depends on the data and the type of solution we are looking for. For instance see (4.31) for the kind of boundary conditions that might arise when we are seeking a particular exact solution. A general statement about the solvability of the equation cannot be made either. If the equation coupled with the boundary conditions (that arise when looking for a specific solution *u*) has a solution then indeed *u* can possibly solve the minimisation problem. On the other hand, if the *p*-Laplace equation does not have a solution then the function *u* that imposed the corresponding boundary conditions cannot be a minimiser. For more details on the *p*-Laplace equation and its solvability we refer the reader to [28] and the references therein.

### Exact Solutions of $(\mathcal {P})$ for a Step Function

In what follows we compute explicit solutions of the $\mathrm {TVL}^{p}$ denoising model $(\mathcal {P})$ for the case $p=2$ for a simple data function. We define the step function in $\Omega =(-L,L)$, $L>0$ as

4.22 $$\begin{aligned} f(x)= {\left\{ \begin{array}{ll} 0&{}\text { if }x\in (-L,0],\\ h&{}\text { if }x\in (0,L). \end{array}\right. } \end{aligned}$$

We first investigate conditions under which we obtain ROF type solutions, that is $w=0$.

#### ROF Type Solutions

We note that in this case we can derive conditions for every $1<p\le \infty$. We are initially interested in solutions that respect the discontinuity at $x=0$ and are piecewise constant. From the optimality conditions (4.11)–(4.12), it suffices to find a function $\phi \in \mathrm {H}^{1}_{0}(\Omega )$ which, apart from $\phi (-L)=\phi (L)=0,$ it also satisfies4.23and it is piecewise affine. It is easy to see that by setting $\phi (x)=\frac{\alpha }{L}(L-|x|)$, the conditions (4.23) are satisfied and the solution *u* is piecewise constant. The first condition of (4.12) implies that  and provides a necessary and sufficient condition that needs to be fulfilled in order for *u* to be piecewise constant, that is to say

4.24 $$\begin{aligned} u(x)= {\left\{ \begin{array}{ll} \frac{\alpha }{L}&{} \text{ if } x\in (-L,0],\\ h-\frac{\alpha }{L}&{} \text{ if } x\in (0,L) \end{array}\right. } \quad \Leftrightarrow \quad \frac{\beta }{\alpha }\ge \left( \frac{2L}{q+1}\right) ^{\frac{1}{q}}. \end{aligned}$$

A special case of the ROF type solution is when *u* is constant, i.e. when $u=\tilde{f}$, the mean value of *f*. We define $\phi (x)=\frac{h}{2}(L-|x|)$ and in that case we have that  and . This implies that

4.25 $$\begin{aligned} u=\tilde{f}=\frac{h}{2}\quad \Leftrightarrow \quad \alpha \ge \frac{hL}{2}\quad \text{ and }\quad \beta \ge \frac{h}{2}\left( \frac{2L^{q+1}}{q+1}\right) ^{\frac{1}{q}}. \end{aligned}$$

Using now (4.24)–(4.25) we can draw the exact regions in the quadrant of $\{\alpha >0,\beta >0\}$ that correspond to these two types of solutions, see the left graph in Fig. 4 for the special case $p=2$. Notice that in these regions $w=0$.

#### $\mathrm {TVL}^2$ Type Solutions

For simplicity reasons, we examine here only the case $p=2$ with $w\ne 0$ in $\Omega$. However, we refer the reader to Sect. 6.2 where we compute numerically solutions for $p\ne 2$. Using Proposition 4.9, we observe that the solution is given by the following second-order differential equation:4.26Even though we can tell that the solution of (4.26) has an exponential form, the fact that the constraint on *C* depends on the solution *w*, creates a difficult computation in order to recover *u* analytically. In order to overcome this obstacle, we consider the one-dimensional version of the 2-homogeneous analogue of $(\mathcal {P})$ that was introduced in Sect. 3:4.27One can derive the optimality conditions for (4.27) similarly to Sect. 4.1. A pair (*w*, *u*) is a solution of (4.27) if and only if there exists a function $\phi \in \mathrm {H}^{1}_{0}(\Omega )$ such that

4.28 $$\begin{aligned} \begin{aligned} \phi '&=u-f, \\ \phi&\in \alpha \mathrm{Sgn}(Du-w),\\ \phi&=\beta _{2-hom}w. \end{aligned} \end{aligned}$$

In view of Proposition 3.2, in order to recover analytically the solutions of $(\mathcal {P})$ for $p=2$ and determine exactly the purple region in Fig. 2, it suffices to solve the equivalent model (4.27) in which $w\ne 0$ holds always. We may restrict our computations only on $(-L,0]\subset \Omega$ and due to symmetry the solution in (0, *L*) is given by $u(x)=h-u(-x)$. The optimality condition (4.28) results to

4.29 $$\begin{aligned} -u''(x)\,\,+\,\,ku(x)=0,\quad \text{ where } \quad k^2=\frac{1}{\beta }\quad \text{ and }\quad x\in (-L,0]. \end{aligned}$$

Then, we get $u(x)=c_{1}e^{kx}+c_{2}e^{-kx}$ with $\phi (x)=\frac{c_{1}}{k}e^{kx}-\frac{c_{2}}{k}e^{-kx}+c_{3}$ for all $x\in (-L,0]$. Firstly, we examine solutions that are continuous which due to symmetry must satisfy $u(0)=\frac{h}{2}$. Since $\phi \in \mathrm {H}^{1}_{0}(-L,L)$, we have $\phi (-L)=0$ and also $u'(-L)=0$. Finally, we require that $\phi (0)<\alpha$. After some computations, we conclude that

4.30 $$\begin{aligned} u(x)= {\left\{ \begin{array}{ll} c_{1}e^{kx}+c_{2}e^{-kx} &{} \text{ if } x\in (-L,0],\\ h-c_{1}e^{-kx}-c_{2}e^{kx}&{} \text{ if } x\in (0,L) \end{array}\right. }\,\, \Leftrightarrow \,\,\frac{\tanh (kL)}{k}<\frac{2\alpha }{h}, \end{aligned}$$

where $$c_{1}=c_{2}e^{2kL}$$, $$c_{2}=\frac{h}{2(e^{2kL}+1)}$$ and $$k=\frac{1}{\sqrt{\beta }}$$.

On the other hand, in order to get solutions that preserve the discontinuity at $x=0$, we require the following:

4.31 $$\begin{aligned} \begin{aligned}&\phi (-L)=0,\quad u'(-L)=0,\\&u(0)<\frac{h}{2},\quad \phi (0)=\alpha . \end{aligned} \end{aligned}$$

Then we get

**Fig. 3:**
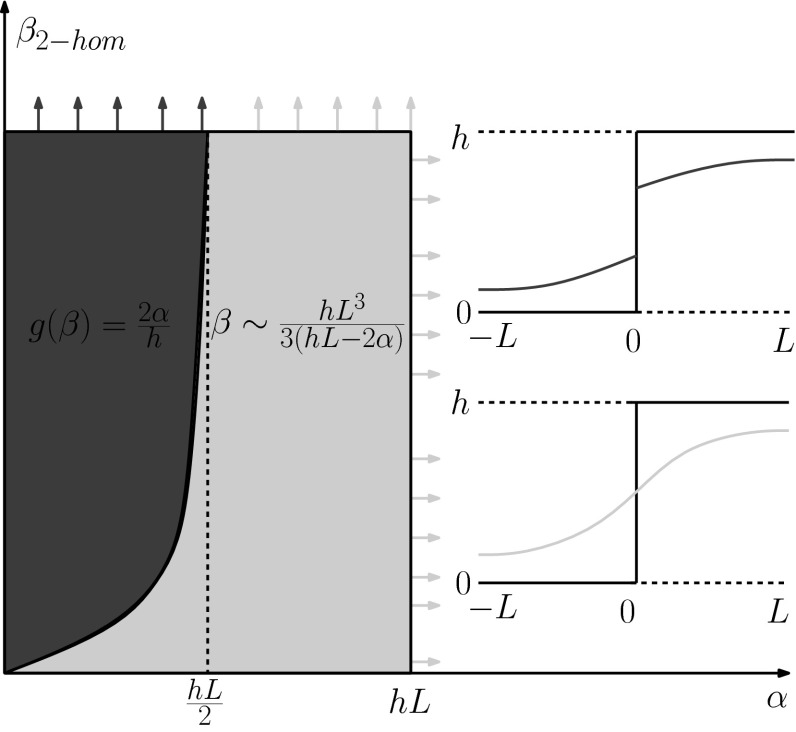
Characterisation of solutions of (4.27) for data *f* being a step function. The *green region* corresponds to solutions that preserve the discontinuity at $x=0$, (4.32), while the *blue region* corresponds to continuous solutions, (4.30), both having an exponential form (Color figure online)

4.32 $$\begin{aligned} u(x)= {\left\{ \begin{array}{ll} c_{1}e^{kx}+c_{2}e^{-kx} &{} \text{ if } x\in (-L,0],\\ h-c_{1}e^{-kx}-c_{2}e^{kx}&{} \text{ if } x\in (0,L) \end{array}\right. }\,\, \Leftrightarrow \,\,\frac{\tanh (kL)}{k}>\frac{2\alpha }{h}, \end{aligned}$$

where $c_{1}=c_{2}e^{2kL}$, $c_{2}=\frac{\alpha k}{e^{2kL}-1}$ and $k=\frac{1}{\sqrt{\beta }}$. Notice that the conditions for $\alpha$ and $\beta$ in (4.30) and (4.32) are supplementary and thus only these type of solutions can occur, see the quadrant of $\{\alpha >0, \beta >0\}$ as it presented in Fig. 3. Letting $g(\beta )=\sqrt{\beta }\tanh {(\frac{L}{\sqrt{\beta }})}$, if $g(\beta )<\frac{2\alpha }{h}$ then the solution is of the form (4.30), see the blue region in Fig. 3. On the other hand in the complementary green region we obtain the solution (4.32). For extreme cases where $\beta \rightarrow \infty$, i.e. $k\rightarrow 0$ we obtain $\frac{\tanh (kL)}{k}\rightarrow L$, which means that there is an asymptote of *g* at $\alpha =\frac{hL}{2}$. Although, we know the form of the inverse function of the hyperbolic tangent, we cannot compute analytically the inverse $f^{-1}$. However, we can obtain an approximation using a Taylor expansion which leads to

4.33 $$\begin{aligned}&\sqrt{\beta }\tanh {\left( \frac{L}{\sqrt{\beta }}\right) }=L - \frac{L^3}{3\beta } + \mathcal {O}\left( \frac{1}{\beta ^2}\right) =\frac{2\alpha }{h}\Leftrightarrow \nonumber \\&\quad \beta =\frac{hL^3}{3(hL-2\alpha )}, \end{aligned}$$

where $$\alpha >0$$ and $$\alpha \ne \frac{hL}{2}$$.

Finally, we would like to describe the solution on the limiting case $\beta \rightarrow \infty$. Letting $\beta \rightarrow \infty$ in (4.30), we have that $c_{1},c_{2}\rightarrow \frac{h}{2}$ and $u(x)\rightarrow \frac{h}{2}$ for every $x\in \Omega$, which in fact is the mean value solution of $(\mathcal {P})$. For the discontinuous solutions, we have that $c_{1},c_{2}\rightarrow \frac{\alpha }{2L}$ and

$$\begin{aligned} u(x)\rightarrow {\left\{ \begin{array}{ll} \frac{\alpha }{L}&{} \text{ if } x\in (-L,0],\\ h-\frac{\alpha }{L}&{} \text{ if } x\in (0,L),\\ \end{array}\right. } \end{aligned}$$

i.e. the limiting solution is (4.24). We also get that

**Fig. 4:**
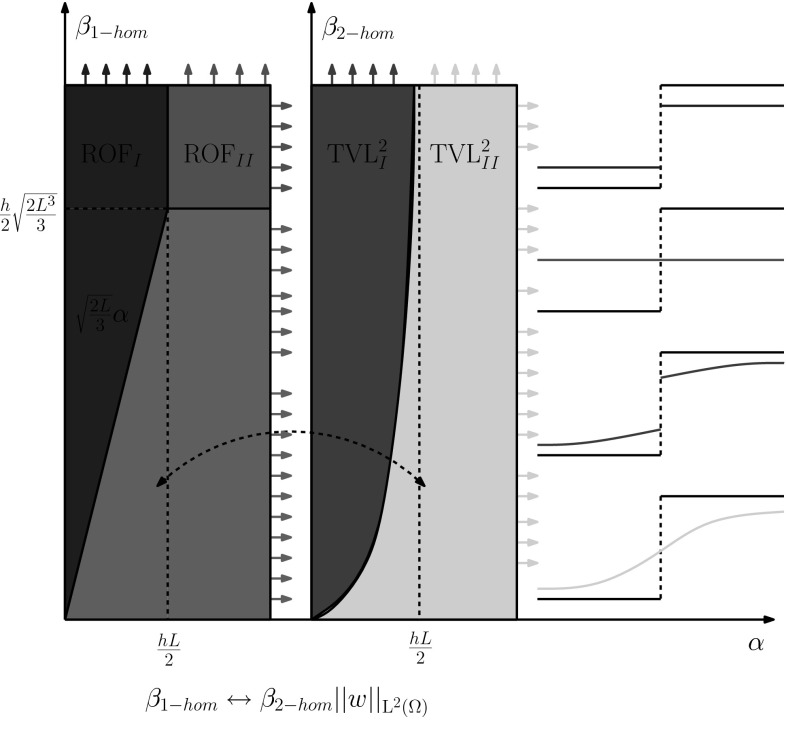
Characterisation of solutions of $(\mathcal {P})$ for $p=2$ for data *f* being a step function. The type of solutions in the *purple region* of the *left graph* are exactly the solutions obtained for the 2-homogeneous problem (4.27), on the *right graph* (Color figure online)

4.34 $$\begin{aligned} w(x)=kc_{2} {\left\{ \begin{array}{ll} e^{2kL+kx}-e^{-kx}&{} \text{ if } x\in (-L,0],\\ e^{2kL-kx}-e^{kx}&{} \text{ if } x\in (0,L],\\ \end{array}\right. } \end{aligned}$$

with  and $c_{2}$ is given either from (4.30) or (4.32). Then, in both cases we have $w\rightarrow 0$ as $k\rightarrow 0$. Observe that the product of  is bounded as $\beta _{2-hom}\rightarrow \infty$ for both types of solutions and in fact corresponds to the bounds found in (4.24) and (4.25). Indeed, since

$$\begin{aligned} \frac{(\sinh (2kL)-2kL)^{\frac{1}{2}}}{k^{\frac{3}{2}}}\rightarrow 2\sqrt{\frac{L^3}{3}},\quad \text{ as } k\rightarrow 0, \end{aligned}$$

if $\alpha >\frac{hL}{2}$ thenwhile if $\alpha \le \frac{hL}{2}$The last result is yet another verification of Proposition 3.2 and it shows that there is an one to one correspondence between  and . The solutions that belong to the purple region of Fig. 4 are the same to the solutions that are shown in Fig. 3.

In the next proposition we summarise the type of solutions for $(\mathcal {P})$ for the step function:

##### **Proposition 4.10**

There are four different types of solutions for the problem $(\mathcal {P})$ with $p=2$ taking the step function (4.22) as data:

1. A piecewise constant solution given in (4.24) (blue region in Fig. 4).
2. A constant solution, equal to the mean value of the data, given in (4.25) (brown region in Fig. 4).
3. A continuous exponential solution given in (4.30) (lightblue region in Fig. 4).
4. A discontinuous piecewise exponential solution given in (4.32) (green region in Fig. 4).

Furthermore, there is an one to one correspondence between the purple and the green/light blue regions in Fig. 4.

## An Image Decomposition Approach

In this section, we present another formulation of the problem $(\mathcal {P})$, where we decompose an image into a $\mathrm {BV}$ part (piecewise constant) and a part that belongs to $W^{1,p}(\Omega )$ (smooth). Let $1<p\le \infty$ and $\Omega \subset \mathbb {R}^{d}$ and consider the following minimisation problem:5.1In this way, we can decompose our image into two components of different structure. The second term captures the piecewise constant structures in the image, whereas the third term captures the smoothness that depends on the value of *p*. In the one-dimensional setting, we can prove that the problems $(\mathcal {P})$ and (5.1) are equivalent.

### **Proposition 5.1**

Let $\Omega =(a,b)\subset \mathbb {R}$ and $1<p\le \infty$. Then a pair $(v^{*},u^{*})\in \mathrm {W}^{1,p}(\Omega )\times \mathrm {BV}(\Omega )$ is a solution of (5.1) if and only if $(\nabla v^{*}, u^{*}+v^{*})\in \mathrm {L}^p(\Omega )\times \mathrm {BV}(\Omega )$ is a solution of $(\mathcal {P})$.

### *Proof*

Let $\overline{u}=u+v$ then, we have the followingHowever, the constraints $w=\nabla v$, $v\in \mathrm {W}^{1,p}(\Omega )$ can simply be substituted by $w\in \mathrm {L}^{p}(\Omega )$ since

5.2 $$\begin{aligned} \left\{ w\in \mathrm {L}^{p}(\Omega ): \;\exists v\in \mathrm {W}^{1,p}(\Omega ),\; w=\nabla v\right\} =\mathrm {L}^{p}(\Omega ). \end{aligned}$$

Indeed, let $w\in \mathrm {L}^{p}(\Omega )\subset \mathrm {L}^{1}(\Omega )$ for $p\in (1,\infty )$ and define $v(x)=\int _{a}^{x}w(s)\,ds$ for $x\in \Omega \subset \mathbb {R}$. Clearly, $v'=w$ a.e. and by Hölder’s inequality we have for every $x\in (a,b)$

$$\begin{aligned} |v(x)|^{p}= & {} \left| \int _{a}^{x}w(s)\,ds\right| ^{p}\le (x-a)^{p-1}\\&\int _{a}^{x}|w(s)|^{p}\,ds <C<\infty . \end{aligned}$$

Thus $v\in \mathrm {W}^{1,p}(\Omega )$ for $p\in (1,\infty )$. If $p=\infty$, suppose again $w\in \mathrm {L}^{\infty }(\Omega )$ and let $C>0$ be a constant such that $|w(x)|\le C$ a.e. on $\Omega$. In that case we have $|v(x)|\le \int _{a}^{x}|w(s)|\,ds\le C|\Omega |<\infty$, i.e. $v\in \mathrm {L}^{\infty }(\Omega )$ and hence $v\in \mathrm {W}^{1,\infty }(\Omega )$ since $v'=w$. Therefore,where $\overline{u^{*}}=u^{*}+v^{*}$ and $w^{*}=\nabla v^{*}$.

Even though for $d=1$ it is true that every $\mathrm {L}^{p}$ function can be written as a gradient, this is not true in higher dimensions. In fact, as we show in the following sections, this constraint is quite restrictive and for example the staircasing effect cannot be always eliminated in the denoising process, see for instance Fig. 20.

The existence of minimisers of (5.1) is shown following again the same techniques as in Theorem 2.5. Moreover, due to the strict convexity of the fidelity term in (5.1), one can prove that the sum $u+v\in \mathrm {BV(\Omega )}$ is unique for a solution $(u,v)\in \mathrm {W}^{1,p}(\Omega )\times \mathrm {BV}(\Omega )$. This result reflects the uniqueness for the problem $(\mathcal {P})$ for $\overline{u}$. However one cannot show that the solutions (*u*, *v*) are unique in general. Yet, one can say something more about this issue. if $(u_{1},v_{1}), (u_{2},v_{2})$ are two minimisers of (5.1), then from the convexity of *L*(*u*, *v*) we have for $0\le \lambda \le 1$

$$\begin{aligned}&L(\lambda (u_{1},v_{1})+(1-\lambda )(u_{2},v_{2}))\le \lambda L(u_{1},v_{1})\\&\quad +\,(1-\lambda )L(u_{2},v_{2}). \end{aligned}$$

Since $(u_{1},v_{1}), (u_{2},v_{2})$ are both minimisers, the above inequality is in fact an equality. Since $u_{1}+v_{1}=u_{2}+v_{2}$, we obtain5.3If we assume thatthen we contradict the equality on (5.3). Hence, the Minkowski inequality becomes an equality, i.e.which is equivalent to the existence of $\mu \ge 0$ such that $\nabla v_{2}=\mu \nabla v_{1}$. In other words, we have proved the following proposition which was also shown in [25] in a similar context:

### **Proposition 5.2**

Let $(u_{1},v_{1}), (u_{2},v_{2})$ be two minimisers of (5.1). Then

5.4 $$\begin{aligned}&u_{1}+v_{1} =u_{2}+v_{2},\quad \text {and}\end{aligned}$$

5.5 $$\begin{aligned}&\text {there exists a }\mu \ge 0 \text{ such } \text{ that } \nabla v_{2} =\mu \nabla v_{1}. \end{aligned}$$

## Numerical Experiments

In this section we present our numerical simulations for the problem $(\mathcal {P})$. We begin with the one-dimensional case where we verify numerically the analytical solutions obtained in Sect. 4.3. Through examples we also investigate the type of structures that are promoted for different values of *p*. Finally, we proceed to the two-dimensional case where we focus on image denoising tasks and in particular on the elimination of the staircasing effect.

We start by defining the discretised version of problem $(\mathcal {P})$6.1Here $\mathrm {TVL}_{\alpha ,\beta }^{p}:\mathbb {R}^{n\times m}\rightarrow \mathbb {R}$ is defined as6.2where for $x\in \mathbb {R}^{n\times m}$, we set  and for $x=(x_{1},x_{2})\in (\mathbb {R}^{n\times m})^2$ we define6.3We denote by $\nabla =(\nabla _{1},\nabla _{2})$ the discretised gradient with forward differences and zero Neumann boundary conditions defined as

$$\begin{aligned} (\nabla _{1} u)_{i,j}&= {\left\{ \begin{array}{ll} u(i+1,j)-u(i,j)&{} \text{ if } 1\le i< n,\, 1\le j\le m,\\ 0 &{} \text{ if } i=n,\; 1\le j\le m, \end{array}\right. }\\ (\nabla _{2} u)_{i,j}&= {\left\{ \begin{array}{ll} u(i,j+1)-u(i,j)&{} \text{ if } 1\le i\le n,\; 1\le j< m,\\ 0 &{} \text{ if } 1\le i\le n,\; j=m. \end{array}\right. } \end{aligned}$$

The discrete version of the divergence operator is defined as the adjoint of discrete gradient. That is, for every $w=(w_{1},w_{2})\in (\mathbb {R}^{n\times m})^2$ and $u\in \mathbb {R}^{n\times m}$, we have $\left\langle -\mathrm{div}w,u\right\rangle _{}=\left\langle w,\nabla u\right\rangle _{}$ with

6.4 $$\begin{aligned} \begin{aligned} (\mathrm{div}w)_{i,j}&= {\left\{ \begin{array}{ll} w_{1}(i,j)-w_{1}(i,j-1)&{} \text{ if } 1< j< m,\; 1\le i\le n,\\ w_{1}(i,j) &{} \text{ if } j=1,\; 1\le i\le n,\\ -w_{1}(i,j-1) &{} \text{ if } j=m,\; 1\le i\le n,\ \end{array}\right. }\\&+ {\left\{ \begin{array}{ll} w_{2}(i,j)-w_{2}(i-1,j)&{} \text{ if } 1<i<n,\; 1\le j\le m,\\ w_{2}(i,j) &{} \text{ if } i=1,\; 1\le j\le m,\\ -w_{2}(i-1,j) &{} \text{ if } i=m,\; 1\le j\le m. \end{array}\right. } \end{aligned} \end{aligned}$$

We solve the minimisation problem (6.1) in two ways. The first one is by using the CVX optimisation package with MOSEK solver (interior point methods) [19]. This method is efficient for small–medium scale optimisation problems and thus it is a suitable choice in order to replicate one-dimensional solutions. On the other hand, we prefer to solve large scale two-dimensional versions of (6.1) with the split Bregman method [18] which has been widely used for the fast solution of non-smooth minimisation problems.

### Split Bregman for L$\mathbf {^{2}}$–TVL$\mathbf {^{p}}$

In this section we describe how we adapt the split Bregman algorithm to our discrete model (6.1). We first transform the unconstrained problem (6.1) into a constrained one by setting $z=\nabla u -w$:6.5Replacing the constraint, using a Lagrange multiplier $\lambda$, we obtain the following unconstrained formulation:6.6The Bregman iteration [32], that corresponds to the minimisation (6.6) leads to the following two-step algorithm:

6.7 $$\begin{aligned} (u^{k+1},z^{k+1},w^{k+1})&=\underset{u,z,w}{{\text {argmin}}} \frac{1}{2} \,\left||f-u\right||_{2}^{2}+\alpha \,\left||z\right||_{1} + \beta \,\left||w\right||_{p}\nonumber \\&\qquad + \frac{\lambda }{2}\,\left||b^{k}-z+\nabla u - w\right||_{2}^{2}, \end{aligned}$$

6.8 $$\begin{aligned} b^{k+1}&=b^{k}+z^{k+1}-\nabla u ^{k+1} -w^{k+1}. \end{aligned}$$

Since solving (6.7) at once is a difficult task, we employ a splitting technique and minimise alternatingly for *u*, *z* and *w*. This yields the split Bregman iteration for our method:

6.9 $$\begin{aligned} u^{k+1}&=\underset{u\in \mathbb {R}^{n\times m}}{{\text {argmin}}}\;\frac{1}{2}\,\left||f-u\right||_{2}^2 + \frac{\lambda }{2}\,\left||b^{k}+z^{k}-\nabla u + w^{k}\right||_{2}^{2},\end{aligned}$$

6.10 $$\begin{aligned} z^{k+1}&=\underset{z\in (\mathbb {R}^{n\times m})^2}{{\text {argmin}}}\;\alpha \,\left||z\right||_{1} + \frac{\lambda }{2}\,\left||b^{k}+z-\nabla u^{k+1} + w^{k}\right||_{2}^{2},\end{aligned}$$

6.11 $$\begin{aligned} w^{k+1}&=\underset{w\in (\mathbb {R}^{n\times m})^{2}}{{\text {argmin}}}\;\beta \,\left||w\right||_{p} + \frac{\lambda }{2}\,\left||b^{k}+z^{k+1}-\nabla u^{k+1} + w\right||_{2}^{2},\end{aligned}$$

6.12 $$\begin{aligned} b^{k+1}&=b^{k}+z^{k+1}-\nabla u ^{k+1} -w^{k+1}. \end{aligned}$$

Next, we discuss how we solve each of the subproblems (6.9)–(6.11). The first-order optimality condition of (6.9) results into the following linear system:

6.13 $$\begin{aligned} \underbrace{(I-\lambda \Delta )}_\text {A}u = \underbrace{f - \lambda \mathrm{div}(b^{k} + z^{k} - w^{k})}_\text {c}. \end{aligned}$$

Here *A* is a sparse, symmetric, positive definite and strictly diagonal dominant matrix, thus we can easily solve (6.13) with an iterative solver such as conjugate gradients or Gauss–Seidel. However, due to the zero Neumann boundary conditions, the matrix *A* can be efficiently diagonalised by the two-dimensional discrete cosine transform,

6.14 $$\begin{aligned} A=W_{nm}^{\intercal } D W_{nm}, \end{aligned}$$

where here $W_{nm}$ is the discrete cosine matrix and $D=diag(\mu _{1},\cdots ,\mu _{nm})$ is the diagonal matrix of the eigenvalues of *A*. In that case, *A* has a particular structure of a block symmetric *Toeplitz-plus-Hankel* matrix with *Toeplitz-plus-Hankel* blocks and one can obtain the solution of (6.9) by three operations involving the two-dimensional discrete cosine transform [20] as follows: Firstly, we calculate the eigenvalues of *A* by multiplying (6.14) with $e_{1}=(1,0,\cdots ,0)^{\intercal }$ from both sides and using the fact that $W_{nm}^{\intercal }W_{nm}=W_{nm}W_{nm}^{\intercal }=I_{nm}$, we get

6.15 $$\begin{aligned} D_{i,i}=\frac{[W_{nm}Ae_{1}]_{i}}{[W_{nm}e_{1}]_{i}}, i=1,2,\cdots ,nm. \end{aligned}$$

Then, the solution of (6.9) is computed exactly by

6.16 $$\begin{aligned} u=W_{nm}^{\intercal } D^{-1} W_{nm} c. \end{aligned}$$

The solution of the subproblem (6.10) is obtained in a closed form via the following shrinkage operator:6.17Finally, we discuss the solution of the subproblem (6.11). In the spirit of [40], we solve (6.11) by a fixed-point iteration scheme. Letting $\kappa =\frac{\beta }{\lambda }$ and $\eta =-b^{k}-z^{k+1}+\nabla u^{k+1}$, the first-order optimality condition of (6.11) becomes6.18For given $w^{k}$, we obtain $w^{k+1}$ by the following fixed-point iteration6.19under the convention that $0/0=0$. We can also consider solving the *p*-homogenous analogue $(\mathcal {P}_{p-hom})$, where for certain values of *p*, e.g. $p=2$, we can solve exactly the corresponding version of (6.11), since in that case $w_{i}^{k+1}=\frac{\eta _{i}}{\kappa +1}$. However, we have observed empirically that there is no significant computational difference between these two methods.

**Fig. 5:**
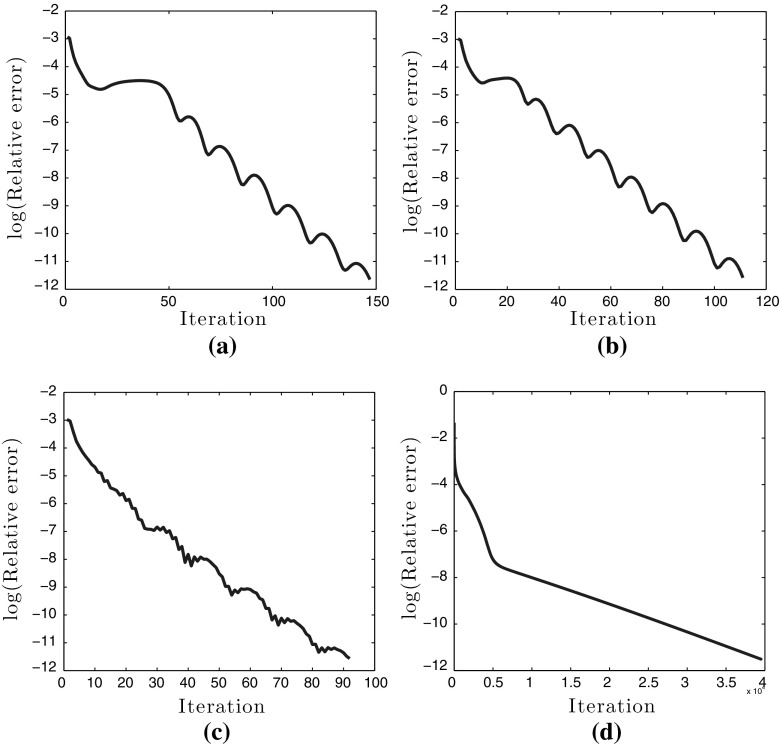
Plots of the relative differences between the split Bregman iterates and the CVX solutions until (6.20) is true with tol $= 10^{-5}$, for the examples in Figs. 13d–f and 14d. In all cases the split Bregman algorithm converges to the solutions given by CVX. **a** Relative residual error between the split Bregman iterates and the CVX solution for the example in Fig. 13d. **b** Relative residual error between the split Bregman iterates and the CVX solution for the example in Fig. 13e. **c** Relative residual error between the split Bregman iterates and the CVX solution for the example in Fig. 13f. **d** Relative residual error between the split Bregman iterates and the CVX solution for the example in Fig. 14d

Since we do not solve all the subproblems (6.9)–(6.11) exactly in every iteration, we cannot guarantee convergence for our version of the split Bregman iteration. Moreover, convergence of the split Bregman algorithm when more than two splittings are performed have not been yet fully established, even though this has been an active field of research lately see for instance [13, 17, 31]. Let us note that the three subproblems in the split Bregman algorithm can be modified into two subproblems (inexact linearised ADMM) with a small cost in the speed of convergence, see for instance [14, 21, 22]. However in practice, the algorithm converges to the right solutions. This claim is supported by the study presented in Fig. 5 where the solutions of the split Bregman iteration are compared to the corresponding solutions obtained with the CVX package for which we have convergence guarantees. There, we have solved the $\mathrm {TVL}^{p}$ minimisations that correspond to Figs. 13d–f and 14d, i.e. for $p=\frac{3}{2},2,3,7$ using both split Bregman and CVX. We plot the relative differences of the split Bregman iterates $u^{k}$ and the CVX solution $u_{\text {CVX}}$ until they are sufficiently close to each other, i.e.6.20In all the plots of Fig. 5, we observe that the split Bregman iterates in practice converge to the CVX solution. Their relative difference becomes of the order $10^{-5}$ in around 100 iterations except for $p=7$, Fig. 5d, where the error tolerance is still reached but only after approximately 10000 iterations.

In Table 1, we compare the computational times of the split Bregman algorithm until (6.20) is satisfied and the computational times of CVX for the same examples as in Fig. 5. The implementations were done in MATLAB (2013) using 2.4 GHz Intel Core 2 Duo and 2 GB of memory. Notice that unless $p=2$, second line in Table 1, CVX needs more than an hour to converge, in contrast to split Bregman where only a few seconds are required for small values of *p*. Note that the split Bregman algorithm is significantly slower for large values of *p*, e.g. $p=7$, see fourth line in Table 1, mainly due to the fixed-point iteration in the subproblem (6.19). We would like to point out that the computational speed can be significantly reduced in the $p=\infty$ case, since the corresponding subproblem is solved exactly, see [8, 36] and in the same time we can obtain similar results to the ones obtained for high values of *p*.

**Table 1:** Computational times of the split Bregman algorithm until (6.20) is true with tol $=10^{-5}$ and comparison to the computational times of CVX for the same examples as in Fig. 5

	Split Bregman (s)	Iterations	Relative error	CVX (s)
$p=1.5$	3.58	147	$8.72\times 10^{-6}$	3433
$p=2$	1.99	111	$9.31\times 10^{-6}$	193.14
$p=3$	1.58	92	$9.49\times 10^{-6}$	3418
$p=7$	2266	39518	$9.68\times 10^{-6}$	3532

### One-Dimensional Results

**Fig. 6:**
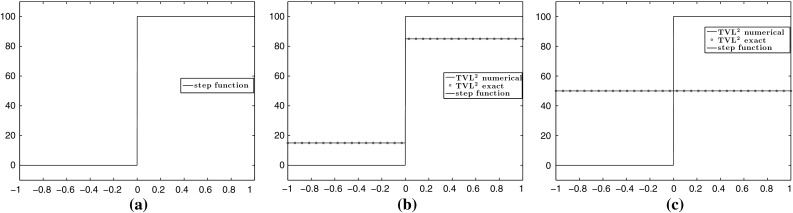
Step function: comparison between numerical solutions of $(\mathcal {P})$ and the corresponding analytical solutions obtained in Sect. 4.3. The parameters $\alpha$ and $\beta$ are chosen so that conditions (4.24) and (4.25) are satisfied which result in ROF solutions. **a** Original data. **b** $\mathrm {TVL}^{2}$: $\alpha =15$, $\beta =500$. **c** $\mathrm {TVL}^{2}$: $\alpha =60$, $\beta =1300$ (Color figure online)

In this section, we present some numerical results in dimension one, i.e. $m=1$, $u\in \mathbb {R}^{n\times 1}$ and $w\in \mathbb {R}^{n\times 1}$. Initially, we compare our numerical solutions with the analytical ones, obtained in Sect. 4.3 for the step function. We set $p=2$, $h=100$, $L=1$ and $\Omega =(-1,1)$ which is discretised into 2000 points. We first examine the cases where ROF solutions are obtained, i.e. the parameters $\alpha$ and $\beta$ are selected according to the conditions (4.24) and (4.25). We have done that in Fig. 6 where we see that the analytical solutions coincide with the numerical ones.

**Fig. 7:**
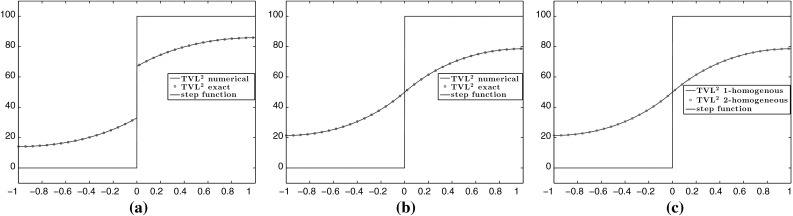
Step function: comparison between numerical and analytical solutions obtained in Sect. 4.3, by solving the 2-homogeneous problem (4.27). The parameters $\alpha$ and $\beta$ are chosen so that conditions (4.30) and (4.32) are satisfied which result in non-ROF solutions. The last plot shows the equivalence between the 1-homogeneous problem $(\mathcal {P})$ and 2-homogeneous (4.27). **a** $\mathrm {TVL^{2}}:$ $\alpha =20$, $\beta _{2-hom}=450$. **b** $\mathrm {TVL^{2}}:$ $\alpha =60$, $\beta _{2-hom}=450$. **c** Equivalence of 1 and 2-homogeneous models: $\alpha =15$, $\beta _{2-hom}$ $=$ 450,  (Color figure online)

We proceed by computing the non-ROF solutions. The numerical solutions are solved using the 2-homogeneous analogue (4.27), since we have proved that the 1-homogeneous and *p*-homogeneous problems are equivalent modulo an appropriate rescaling of the parameter $\beta$, see Proposition 3.2. Indeed, as it is described in Fig. 4, in order to obtain solutions from the purple region, it suffices to seek for solutions of the 2-homogeneous (4.27). Recall also that these solutions are exactly the solutions obtained solving a Huber TV problem, see Proposition 3.3. The analytical solutions are given in (4.30) and (4.32) and are compared to the numerical ones in Fig. 7, where we observe that they coincide. We also verify the equivalence between the 1-homogeneous and 2-homogeneous problems where $\alpha$ is fixed and $\beta$ is obtained from Proposition 3.2, see Fig. 7c.

**Fig. 8:**
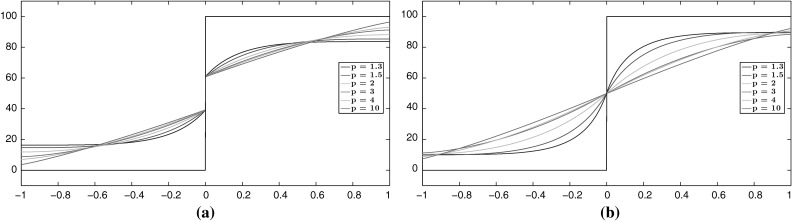
Step function: the types of solutions for the problem $(\mathcal {P})$ for different values of *p*. **a** $\mathrm {TVL}^p$ discontinuous solutions for $p=\{\frac{4}{3}, \frac{3}{2}, 2, 3, 4, 10\}$. **b** $\mathrm {TVL}^p$ continuous solutions for $p=\{\frac{4}{3}, \frac{3}{2}, 2, 3, 4, 10\}$ (Color figure online)

We continue our experiments for general values of *p* focusing on the structure of the solutions as *p* increases. In order to compare the solutions for different values $p\in (1,\infty )$, we fix the parameter $\alpha$ and choose appropriate values of $\beta$. Since we are mainly interested in non-ROF solutions, we choose $\alpha$ and $\beta$ so that they belong to the purple region of Fig. 4, i.e. $\beta <(\frac{2L}{q+1})^{\frac{1}{q}}\alpha$ and $\beta <\frac{h}{2} (\frac{2L^{q+1}}{q+1})^{\frac{1}{q}}$. We set $p=\{\frac{4}{3}, \frac{3}{2}, 2, 3, 4, 10\}$ and in order to get solutions that preserve the discontinuity we set $\beta =\{72, 140, 430, 1350, 2400, 6800\}$ with fixed $\alpha =20$, see Fig. 8a. In order to obtain continuous solutions, we set $\alpha =60$ and $\beta =\{50, 110, 430, 1700, 3000, 9500\}$, see Fig. 8b. We observe that for $p=\frac{4}{3}$, the solution has a similar behaviour to $p=2$, but with a steeper gradient at the discontinuity point and the solution becomes almost constant near the boundary of $\Omega$. On the other hand, as we increase *p*, the slope of the solution near the discontinuity point reduces and it becomes almost linear with a relative small constant part near the boundary.

The linear structure of the solutions that appears for large *p* motivates us to examine the case of piecewise affine data *f* defined as

**Fig. 9:**
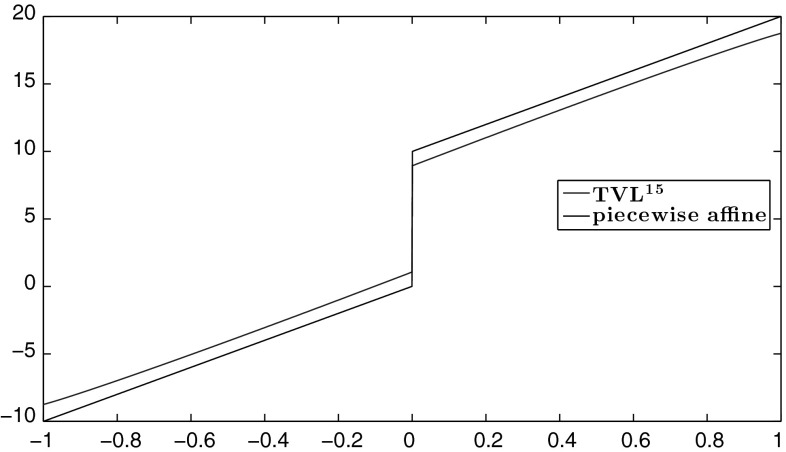
Piecewise affine data: $\mathrm {TVL}^{15}$ solution with $\alpha =1$, $\beta =620$ (Color figure online)

6.21 $$\begin{aligned} f(x)= {\left\{ \begin{array}{ll} \lambda x&{} \text{ if } x\in (-L,0],\\ \lambda x+h&{} \text{ if } x\in (0,L], \end{array}\right. } \end{aligned}$$

see Fig. 9. We set again $\Omega =(-1,1)$ and $\lambda =\frac{1}{10}$. As we observe, the reconstruction for $p=15$ behaves almost linearly everywhere in $\Omega$ except near the boundary. In the follow-up paper [8], where the case $p=\infty$ is examined in detail and exact solutions are computed for the data (6.21), the occurrence of this linear structure is justified also analytically.

**Fig. 10:**
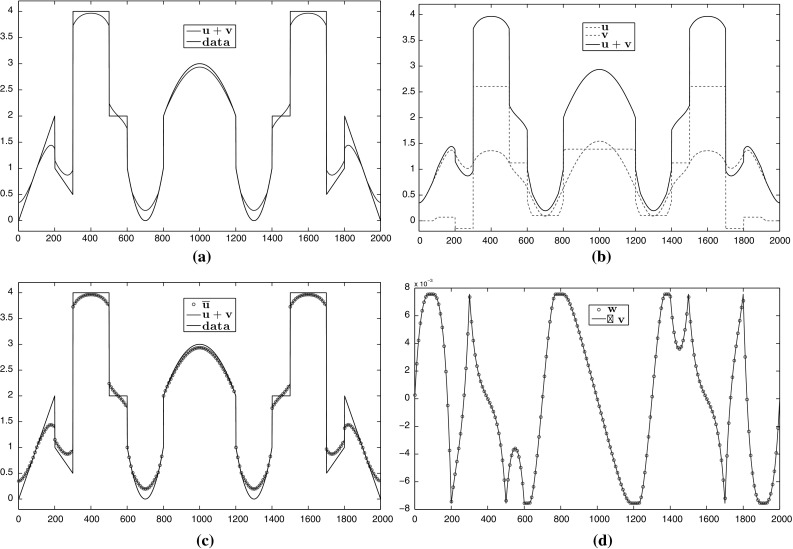
Numerical examples of the image decomposition approach (5.1) for $p=2$. **a** Solution $u+v$ of (5.1). **b** Decomposition into *u*, *v* parts. **c** Equivalence of $(\mathcal {P})$ and (5.1): $\overline{u}=u+v$. **d** Equivalence of $(\mathcal {P})$ and (5.1): $w=\nabla v$ (Color figure online)

**Fig. 11:**
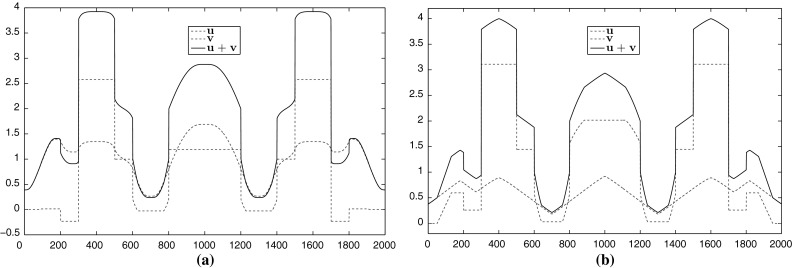
Decomposition of the data in Fig. 10a into *u*, *v* parts for $p=\frac{4}{3}$ and $p=10$. The value $p=\frac{4}{3}$ produces a *v* component with flat structures while $p=10$ produces a component with almost affine structures. In both cases we have . **a** Decomposition of the data in Fig. 10a for $p=\frac{4}{3}$. **b** Decomposition of the data in Fig. 10a for $p=10$ (Color figure online)

In the last part of this section, we present some numerical examples of the image decomposition approach presented in Sect. 5. We use as data a more complicated one-dimensional noiseless signal with piecewise constant, affine and quadratic components and solve the discretised version of (5.1) using $\mathrm {CVX}$. In Fig. 10, we verify numerically the equivalence between (5.1) and $(\mathcal {P})$ for $p=2$, i.e. we show that $(\nabla v,u+v)$ corresponds to $(w,\overline{u})$ where (*v*, *u*) and $(w,\overline{u})$ are the solutions of (5.1) and $(\mathcal {P})$, respectively. We also compare the decomposed parts *u*, *v* for $p=\frac{4}{3}$ and $p=10$. In order to have a reasonable comparison on the corresponding solutions, the parameters $\alpha , \beta$ are selected such that the residual  is the same for both values of *p*. As we observe, the *v* decomposition with $p=\frac{4}{3}$ exhibits some *flatness* compared to $p=2$, compare Figs. 10b and 11a. On the other hand for $p=10$, the *v* component consists again of almost affine structures, Fig. 11b. Notice, that in both cases the *v* components are continuous. This is expected since in dimension one, we have $\mathrm {W}^{1,p}(\Omega )\subset C(\overline{\Omega })$ for every $1<p<\infty$.

### Two-Dimensional Results

In this section we consider the two-dimensional case where $u\in \mathbb {R}^{n\times m}$, $w\in (\mathbb {R}^{n\times m})^2$ with $m>1$ and $\Omega$ is a rectangular image domain. We focus on image denoising tasks and on eliminating the staircasing effect for different values of *p*.

**Fig. 12:**
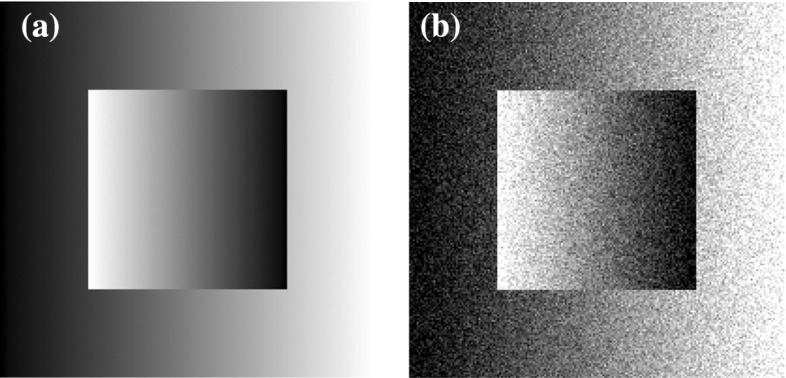
Square with piecewise affine structures and its noisy version with $\sigma =0.01$. **a** Square. **b** Noisy square: PSNR $=$ 20.66 and SSIM $=$ 0.1791

We start with the image in Fig. 12, i.e. a square with piecewise affine structures. The image size is $200\times 200$ pixels at a [0, 1] intensity range. The noisy image, Fig. 12b, is a corrupted version of the original image, Fig. 12a, with Gaussian noise of zero mean and standard deviation $\sigma =0.01$.

**Fig. 13:**
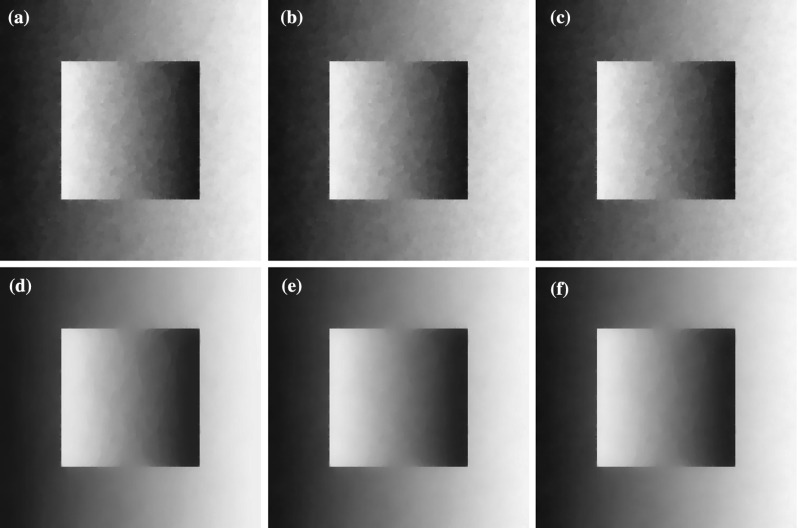
Best reconstructions in terms of PSNR and SSIM for $p=\frac{3}{2}$, 2, 3. **a** $\mathrm {TVL}^{\frac{3}{2}}$: $\alpha \,=\,0.1$, $\beta =2.5$, PSNR $=$ 33.63. **b** $\mathrm {TVL}^{2}$: $\alpha \,=\,0.1$, $\beta =13.5$, PSNR $=$ 33.68. **c** $\mathrm {TVL}^{3}$: $\alpha \,=\,0.1$, $\beta =76$, PSNR $=$ 33.70. **d** $\mathrm {TVL}^{\frac{3}{2}}$: $\alpha =0.3$, $\beta =7.7$, SSIM $=$ 0.9669. **e** $\mathrm {TVL}^{2}$: $\alpha =0.3$, $\beta =34$, SSIM $=$ 0.9706. **f** $\mathrm {TVL}^{3}$: $\alpha =0.3$, $\beta =182$, SSIM $=$ 0.9709

In Fig. 13, we present the best reconstructions results in terms of two quality measures, the classical *Peak Signal to Noise Ratio* (PSNR) and the *Structural Similarity Index* (SSIM), see [41] for the definition of the latter. In each case, the values of $\alpha$ and $\beta$ are selected appropriately for optimal PSNR and SSIM. We use here the split Bregman algorithm as this is described in Sect. 6.1. Our stopping criterion is the relative residual error becoming less than $10^{-6}$, i.e.6.22For computational efficiency, we set $\lambda =10\alpha$ when $1<p<4$ and $\lambda =1000\alpha$ when $p\ge 4$ (empirical rule).

Observe that the best reconstructions in terms of the PSNR have no visual difference for $p=\frac{3}{2}$, 2 and 3 and staircasing is present, Fig. 13a–c. This is one more indication that the PSNR—which is based on the squares of the pointwise differences between the ground truth and the reconstruction—does not correspond to the optimal visual results. The best reconstructions in terms of SSIM are visually better. They exhibit significantly reduced staircasing for $p=\frac{3}{2}$ and $p=3$ and is essentially absent in the case of $p=2$, see Fig. 13d–f.

**Fig. 14:**
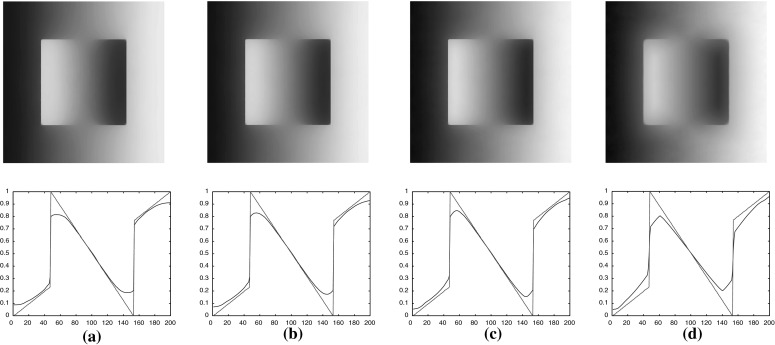
Staircasing elimination for $p=\frac{3}{2}$, 2, 3 and 7. High values of *p* promote almost affine structures. **a** $\mathrm {TVL}^{\frac{3}{2}}$: $\alpha =1$, $\beta =25$, SSIM $=$ 0.9391. **b** $\mathrm {TVL}^{2}$: $\alpha =1$, $\beta =116$, SSIM $=$ 0.9433. **c** $\mathrm {TVL}^{3}$: $\alpha =1$, $\beta =438$, SSIM $=$ 0.9430. **d** $\mathrm {TVL}^{7}$: $\alpha =2$, $\beta =5000$, SSIM $=$ 0.9001

We can also get a total staircasing elimination by setting higher values for the parameters $\alpha$ and $\beta$, as we show in Fig. 14. There, one observes that on one hand as we increase *p*, almost affine structures are promoted—see the middle row profiles in Fig. 14—and on the other hand these choices of $\alpha , \beta$ produce a serious loss of contrast that however can be easily treated via the *Bregman iteration* that we briefly discuss next.

Contrast enhancement via Bregman iteration was introduced in [32], see also [3] for an application to higher-order models. It involves solving a modified version of the minimisation problem. Setting $u^{0}=f$, one solves for $k=1,2,\ldots$:6.23Instead of solving (6.1) once for fixed $\alpha$ and $\beta$, we solve a sequence of similar problems adding back a noisy residual in each iteration. For stopping criteria regarding the Bregman iteration we refer to [32].

**Fig. 15:**
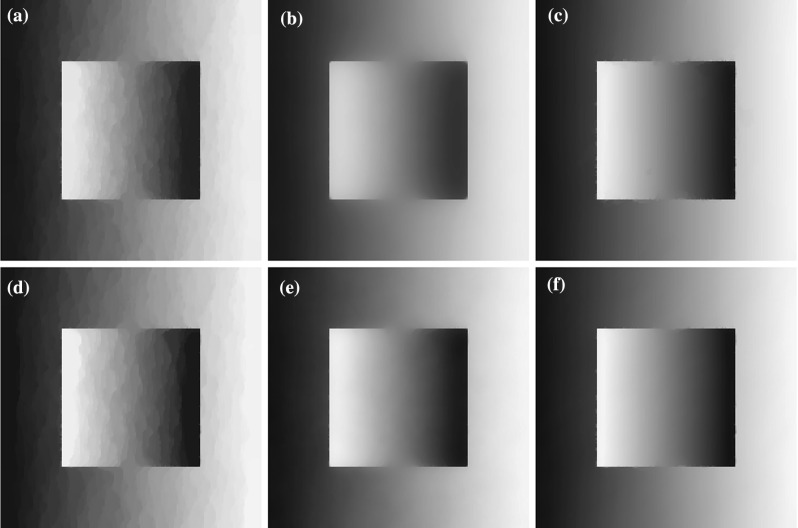
*First row* Best reconstructions in terms of SSIM for $\mathrm {TV}$, $\mathrm {TVL}^{2}$ and $\mathrm {TGV}$. *Second row* Best reconstructions in terms of SSIM for the Bregman iteration versions of $\mathrm {TV}$, $\mathrm {TVL}^{2}$ and $\mathrm {TGV}$. **a** $\mathrm {TV}$: $\alpha =0.2$, SSIM $=$ 0.9387. **b** $\mathrm {TVL}^{2}$: $\alpha =1$, $\beta =116$, SSIM $=$ 0.9433. **c** $\mathrm {TGV}$: $\alpha =0.12$, $\beta =0.55$, SSIM $=$ 0.9861. **d** $\mathrm {TV}$ Bregman iteration: $\alpha =1$, SSIM $=$ 0.9401, 4th iteration. **e** $\mathrm {TVL}^{2}$ Bregman iteration: $\alpha =2$, $\beta =220$, SSIM $=$ 0.9778, 4th iteration. **f** $\mathrm {TGV}$ Bregman iteration: $\alpha =2$, $\beta =10$, SSIM $=$ 0.9889, 8th iteration

In Fig. 15 we present our best Bregman iteration results for $p=2$ in terms of SSIM along with the corresponding $\mathrm {TV}$ and $\mathrm {TGV}$ results for which the Bregman iteration has also been employed for the sake of fair comparison. We also show the best SSIM results where no Bregman iteration is used. We solve the $\mathrm {TGV}$ minimisation using the Chambolle–Pock primal-dual method [10]. We notice that the Bregman iteration version of $\mathrm {TVL}^{2}$ leads to a significant contrast improvement, compare for instance Fig. 15b, e. In fact, it can achieve a reconstruction which is visually close to the Bregman iteration version of $\mathrm {TGV}$, compare Fig. 15e, f.

**Fig. 16:**
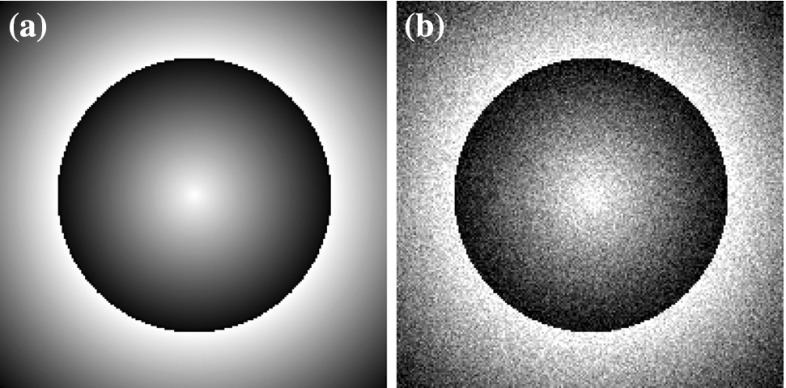
Image with symmetric radial structures and its noisy version with $\sigma =0.01$. **a** Circle. **b** Noisy circle: SSIM $=$ 0.2457

**Fig. 17:**
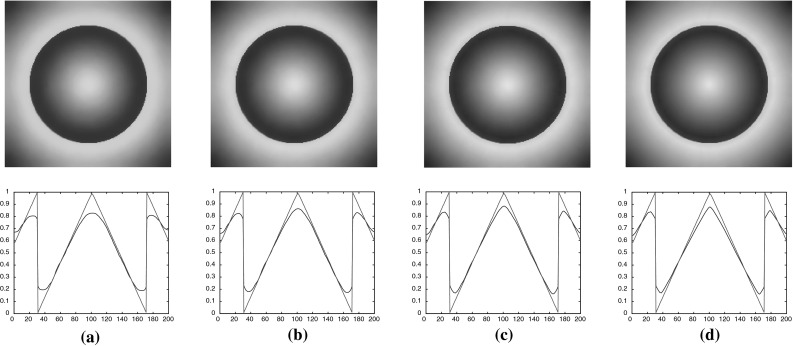
Better preservation of spike-like structures for large values of *p*. **a** $\mathrm {TVL}^{\frac{3}{2}}$: $\alpha =0.8$, $\beta =17$, SSIM $=$ 0.8909. **b** $\mathrm {TVL}^{2}$: $\alpha =0.8$, $\beta =79$, SSIM $=$ 0.8998. **c** $\mathrm {TVL}^{3}$: $\alpha =0.8$, $\beta =405$, SSIM $=$ 0.9019. **d** $\mathrm {TVL}^{7}$: $\alpha =0.8$, $\beta =3700$, SSIM $=$ 0.9024

**Fig. 18:**
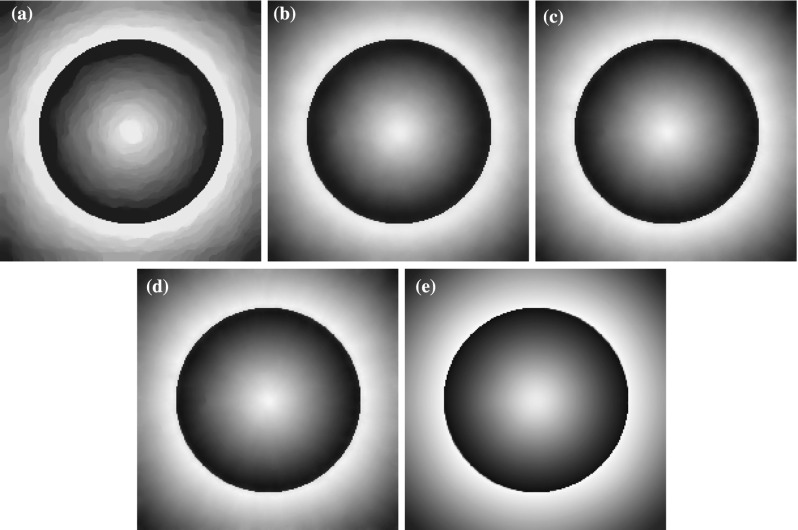
Best reconstruction in terms of SSIM for the Bregman iteration versions of $\mathrm {TVL}^{2}$, $\mathrm {TVL}^{4}$, $\mathrm {TVL}^{7}$ and $\mathrm {TGV}$. **a** $\mathrm {TV}$ Bregman iteration: $\alpha =2$, SSIM $=$ 0.8912, 6th iteration. **b** $\mathrm {TVL^{2}}$ Bregman iteration: $\alpha =5$, $\beta =625$, SSIM $=$ 0.9718, 12th iteration. **c** $\mathrm {TVL^{4}}$ Bregman iteration: $\alpha =5$, $\beta =8000$, SSIM=0.9802, 13th iteration. **d** $\mathrm {TVL^{7}}$ Bregman iteration: $\alpha =3$, $\beta =15000$, SSIM $=$ 0.9807, 9th iteration. **e** $\mathrm {TGV}$ Bregman iteration: $\alpha =2$, $\beta =10$, SSIM $=$ 0.9913, 8th iteration

**Fig. 19:**
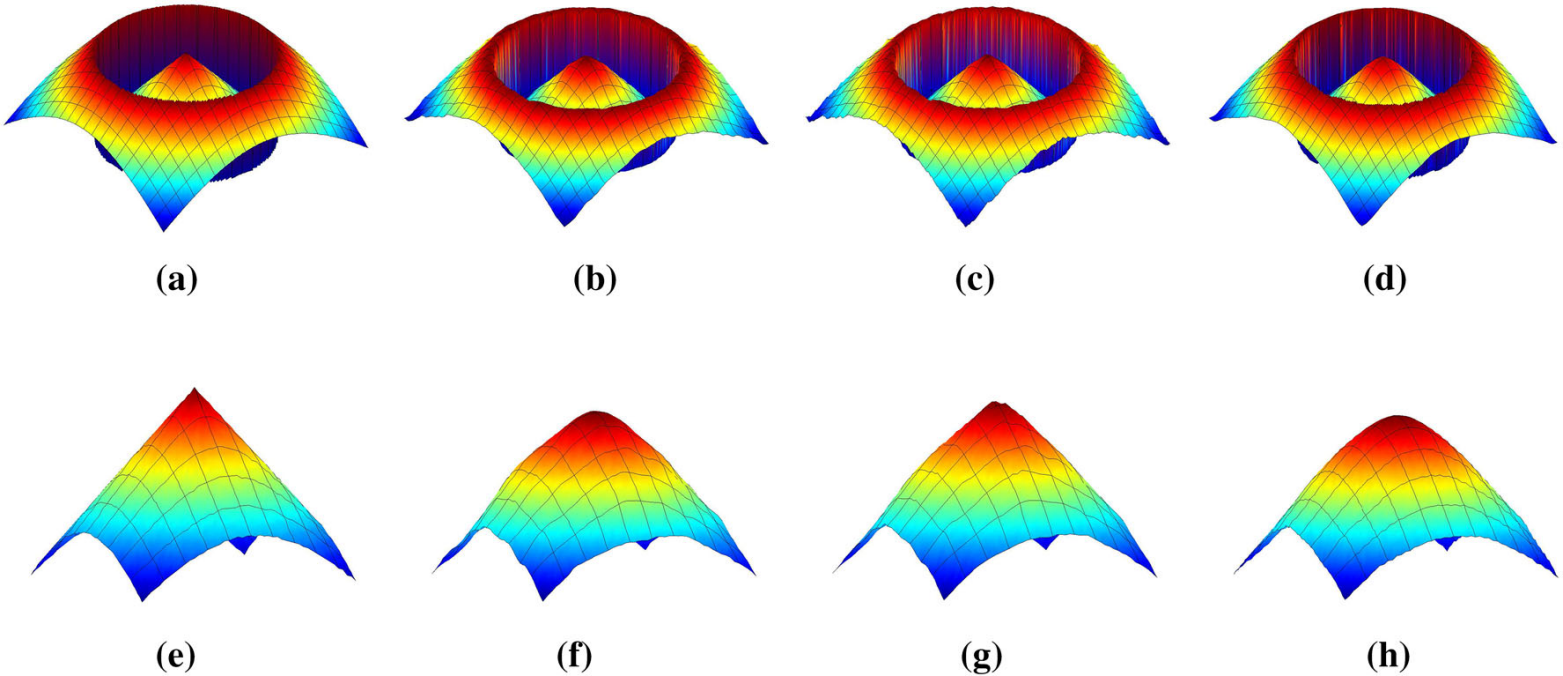
Surface plots of the images in Fig. 18. Notice how high values of *p* can better preserve the sharp spike in the middle of the image. **a** Original. **b** $\mathrm {TVL}^{2}$ Bregman iteration. **c** $\mathrm {TVL}^{7}$ Bregman iteration. **d** $\mathrm {TGV}$ Bregman iteration. (e) Original: central part zoom. **f** $\mathrm {TVL}^{2}$ Bregman iteration: central part zoom. **g** $\mathrm {TVL}^{7}$ Bregman iteration: central part zoom. **h** $\mathrm {TGV}$ Bregman iteration: central part zoom (Color figure online)

We continue our demonstration with a radially symmetric image, see Fig. 16. As in the previous example, we can achieve staircasing-free reconstructions using $\mathrm {TVL}^{p}$ regularisation for different values of *p*, see Fig. 17. In fact, as we increase *p*, we obtain results that preserve the spike in the centre of the circle, see the corresponding middle row slice in Fig. 17d. This provides us with another motivation to examine the $p=\infty$ case in [8]. The loss of contrast can be again treated using the Bregman iteration (6.23). The best results of the latter in terms of SSIM are presented in Fig. 18, for $p=2$, 4 and 7 and they are also compared to the corresponding Bregman iteration version of $\mathrm {TGV}$. We observe that we can obtain reconstructions that are visually close to the $\mathrm {TGV}$ ones and in fact notice that for $p=7$, the spike on the centre of the circle is better reconstructed compared to $\mathrm {TGV}$, see also the surface plots in Fig. 19.

**Fig. 20:**
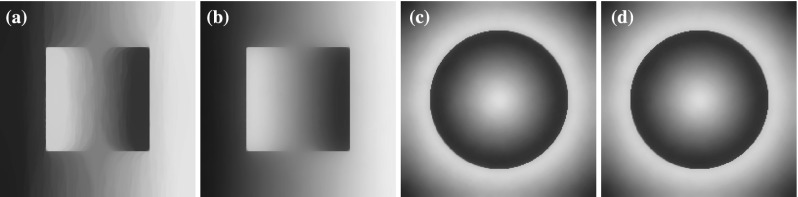
Comparison between the model (5.1) for $p=2$ and $\mathrm {TVL^{2}}$: Staircasing cannot be always eliminated by using the decomposition approach (5.1). **a** Solution $u+v$ of (5.1): $p=2$, $\alpha =0.8$, $\beta =120$, SSIM $=$ 0.9268. **b** $\mathrm {TVL}^{2}$: $\alpha =1$, $\beta =116$, SSIM $=$ 0.9433. **c** Solution $u+v$ of (5.1): $p=2$, $\alpha \,=\,0.8$, $\beta =70$, SSIM $=$ 0.8994. **d** $\mathrm {TVL^{2}}$: $\alpha =0.8$, $\beta =79$, SSIM $=$ 0.8998

We conclude with numerical results for the image decomposition approach of Sect. 5 which we solve again using the split Bregman algorithm. Recall that in dimension two, the solutions of (5.1) are not necessarily the same with the ones of $(\mathcal {P})$. In fact, we observe that (5.1) cannot always eliminate the staircasing, see for instance Figure 20. Even though, as we have already seen, we can eliminate the staircasing both in the square and in the circle by applying $\mathrm {TVL}^{p}$ regularisation, Fig. 20b, d, we cannot obtain equally satisfactory results by solving (5.1). While using the latter we can get rid of the staircasing in the circle, Fig. 20c, this is not possible for the square, Fig. 20a, where we observe—after extensive experimentation—that no values of $\alpha$ and $\beta$ lead to a staircasing elimination. This is analogous to the difference between $\mathrm {TGV}$ and the $\mathrm {TV}$–$\mathrm {TV}^{2}$ infimal convolution of Chambolle–Lions [9].

**Fig. 21:**
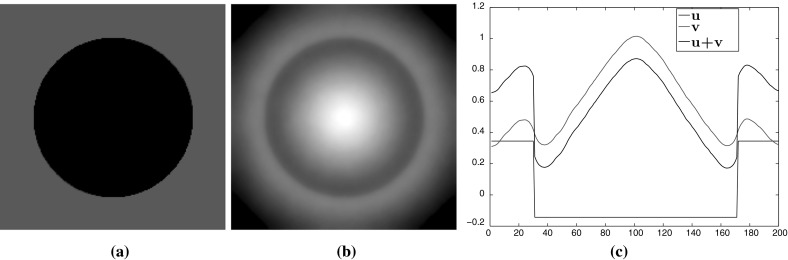
Efficient geometric decomposition of the image in Fig. 20c into a piecewise constant and smooth component, *u* and *v*, respectively, by solving (5.1). **a** Piecewise constant component *u*. **b** Smooth component *v*, **c** Middle row profiles (Color figure online)

However, the strength of the formulation (5.1) lies on its ability to efficiently decompose an image into piecewise constant and smooth parts. We depict that in Fig. 21, where we show the components *u* and *v* of the result in Fig. 20c.

## Conclusion

We have introduced a novel first-order, one-homogeneous $\mathrm {TV}$–$\mathrm {L}^{p}$ infimal convolution type functional suitable for variational image regularisation. The $\mathrm {TVL}^{p}$ functional constitutes a very general class of regularisation functionals exhibiting diverse smoothing properties for different choices of *p*. In the case $p=2$ the well-known Huber $\mathrm {TV}$ regulariser is recovered.

We studied the corresponding one-dimensional denoising problem focusing on the structure of its solutions. We computed exact solutions of this problem for the case $p=2$ for simple one-dimensional data. Hence, as an additional novelty in our paper we presented exact solutions of the one-dimensional Huber $\mathrm {TV}$ denoising problem.

Numerical experiments for several values of *p* indicate that our model leads to an elimination of the staircasing effect. We show that we can further enhance our results by increasing the contrast via a Bregman iteration scheme and thus obtaining results of similar quality to those of $\mathrm {TGV}$. Furthermore, as *p* increases the structure of the solutions changes from piecewise smooth to piecewise linear and the model, in contrast to $\mathrm {TGV}$, is capable of preserving sharp spikes in the reconstruction. This observation motivates a more detailed study of the $\mathrm {TVL}^{p}$ functional for large *p* and in particular for the case $p=\infty$.

This concludes the first part of the study of the $\mathrm {TVL}^{p}$ model for $p< \infty$. The second part [8], is devoted to the $p=\infty$ case. There we explore further, both in an analytical and an experimental level, the capability of the $\mathrm {TVL}^{\infty }$ model to promote affine and spike-like structures in the reconstructed image and we discuss several applications.

## Appendix: Radon Measures and Functions of Bounded Variation

In what follows $$\Omega \subset \mathbb {R}^{d}$$ is an open, bounded set with Lipschitz boundary whose Lebesgue measure is denoted by $$|\Omega |$$. We denote by $$\mathcal {M}(\Omega ,\mathbb {R}^{d})$$ (and $$\mathcal {M}(\Omega )$$ if d $$=$$ 1) the space of finite Radon measures on $$\Omega $$. The total variation measure of $$\mu \in \mathcal {M}(\Omega ,\mathbb {R}^{d})$$ is denoted by $$|\mu |$$, while we denote the polar decomposition of $$\mu $$ by $$\mu =\mathrm {sgn}(\mu )|\mu |$$, where $$\mathrm {sgn}(\mu )=1$$$$|\mu |$$-almost everywhere.

Recall that the *Radon norm* of a $\mathbb {R}^{d}$-valued distribution $\mathcal {T}$ on $\Omega$ is defined as

It can be shown that $\Vert \mathcal {T}\Vert _{\mathcal {M}}<\infty$ if and only if $\mathcal {T}$ can be represented by a measure $\mu \in \mathcal {M}(\Omega ,\mathbb {R}^{d})$ and in that case $\Vert \mu \Vert _{\mathcal {M}}= |\mu |(\Omega )$. Regarding the subdifferential of the Radon norm we have that it can be characterised, at least for $C_{0}$ functions, as follows [6]

8.1

where here $\mathrm{Sgn}(\mu )$ denotes the set-valued sign

8.2

A function $u\in \mathrm {L}^{1}(\Omega )$ is a function of bounded variation if its distributional derivative *Du* is representable by a finite Radon measure. We denote by $\mathrm {BV}(\Omega )$, the space of functions of bounded variation which is a Banach space under the norm

The term

is called the total variation of *u*, also commonly denoted by $\mathrm {TV}(u)$. From the Radon–Nikodym theorem, the measure $\mu$ can be decomposed into an absolutely continuous and a singular part with respect to the Lebesgue measure $\mathcal {L}^{d}$, that is $Du=D^{a}u+D^{s}u$. Here, $D^{a}u=\nabla u\mathcal {L}^{d}$, i.e. $\nabla u$ denotes the Radon–Nikodym derivative of $D^{a}u$ with respect to $\mathcal {L}^{d}$. Note that we also have $\Vert Du\Vert _{\mathcal {M}}=\Vert D^{a}u\Vert _{\mathcal {M}}+\Vert D^{s}u\Vert _{\mathcal {M}}$. When $d=1$, $\nabla u$ is simply denoted by $u'$. Finally, we recall that $\mathrm {BV}(\Omega )\hookrightarrow \mathrm {L}^{r}(\Omega )$ for $1\le r \le \frac{d}{d-1}$ (resp. $1\le r <\frac{d}{d-1}$ ) with continuous embedding (resp. compact embedding) .

Recall also the following basic inequality regarding inclusions of $\mathrm {L}^{p}$ spaces

8.3

Unless otherwise stated *q* denotes the Hölder conjugate of the exponent *p*, i.e.

8.4 $$\begin{aligned} q= {\left\{ \begin{array}{ll} \frac{p}{p-1}&{} \text{ if } p\in (1,\infty ),\\ 1 &{} \text{ if } p=\infty . \end{array}\right. } \end{aligned}$$
